# Prey availability and temporal partitioning modulate felid coexistence in Neotropical forests

**DOI:** 10.1371/journal.pone.0213671

**Published:** 2019-03-12

**Authors:** Fernanda Santos, Chris Carbone, Oliver R. Wearn, J. Marcus Rowcliffe, Santiago Espinosa, Marcela Guimarães Moreira Lima, Jorge A. Ahumada, André Luis Sousa Gonçalves, Leonardo C. Trevelin, Patricia Alvarez-Loayza, Wilson R. Spironello, Patrick A. Jansen, Leandro Juen, Carlos A. Peres

**Affiliations:** 1 Programa de Pós-graduação em Ecologia/Universidade Federal do Pará, Belém, Pará, Brazil; 2 Department of Mastozoology—Museu Paraense Emílio Goeldi, Belém, Pará, Brazil; 3 Institute of Zoology, Zoological Society of London, London, United Kingdom; 4 Universidad Autónoma de San Luis Potosí, San Luis Potosí, Mexico; 5 Escuela de Ciencias Biológicas, Pontificia Universidad Católica del Ecuador, Quito, Ecuador; 6 Laboratório de Ecologia e Conservação/Universidade Federal do Pará, Belém, Pará, Brazil; 7 Moore Center for Science, Conservation International, Arlington, Virginia, United States of America; 8 Grupo de Pesquisas de Mamíferos Amazônicos (GPMA), Instituto Nacional de Pesquisas da Amazônia (INPA), Manaus, Amazonas, Brazil; 9 Center for Tropical Conservation, Duke University, Durham, North Carolina, United States of America; 10 Center for Tropical Forest Science, Smithsonian Tropical Research Institute, Balboa, Ancon, Republic of Panama; 11 Department of Environmental Sciences, Wageningen University, Wageningen, The Netherlands; 12 Centre for Ecology, Evolution and Conservation, School of Environmental Sciences, University of East Anglia, Norwich, United Kingdom; University of Alberta, CANADA

## Abstract

Carnivores have long been used as model organisms to examine mechanisms that allow coexistence among ecologically similar species. Interactions between carnivores, including competition and predation, comprise important processes regulating local community structure and diversity. We use data from an intensive camera-trapping monitoring program across eight Neotropical forest sites to describe the patterns of spatiotemporal organization of a guild of five sympatric cat species: jaguar (*Panthera onca*), puma (*Puma concolor*), ocelot (*Leopardus pardalis)*, jaguarundi (*Herpailurus yagouaroundi*) and margay (*Leopardus wiedii*). For the three largest cat species, we developed multi-stage occupancy models accounting for habitat characteristics (landscape complexity and prey availability) and models accounting for species interactions (occupancy estimates of potential competitor cat species). Patterns of habitat-use were best explained by prey availability, rather than habitat structure or species interactions, with no evidence of negative associations of jaguar on puma and ocelot occupancy or puma on ocelot occupancy. We further explore temporal activity patterns and overlap of all five felid species. We observed a moderate temporal overlap between jaguar, puma and ocelot, with differences in their activity peaks, whereas higher temporal partitioning was observed between jaguarundi and both ocelot and margay. Lastly, we conducted temporal overlap analysis and calculated species activity levels across study sites to explore if shifts in daily activity within species can be explained by varying levels of local competition pressure. Activity patterns of ocelots, jaguarundis and margays were similarly bimodal across sites, but pumas exhibited irregular activity patterns, most likely as a response to jaguar activity. Activity levels were similar among sites and observed differences were unrelated to competition or intraguild killing risk. Our study reveals apparent spatial and temporal partitioning for most of the species pairs analyzed, with prey abundance being more important than species interactions in governing the local occurrence and spatial distribution of Neotropical forest felids.

## Introduction

Species interactions comprise one of the most important processes maintaining the structure of local biological diversity, including how species with similar ecological requirements can coexist [1]. Among various existing interspecific ecological relationships, competitive and predation interactions, and their reciprocal effects, have the potential to affect diversity patterns equally, each of which could either limit or promote coexistence [2].

Following the competitive exclusion principle, if two or more species locally compete for the same limiting resource, then interspecific competition may exclude a particular species from the community, suggesting an upper boundary in the number of species that can be accommodated within a niche space [3,4]. However, competing species can coexist when diverging in their niche space, partitioning one or more niche axes: space, time and food resources [5]. Although, whether the ultimate outcome is either coexistence or exclusion is primarily determined by whether partitioning of the dominant interactions occurs—be that competition or predation [2].

In mammalian communities, carnivore species are a model group to study mechanisms of coexistence, because they occupy higher trophic levels and exhibit greater similarity in morphology and ecological requirements [6–9]. Niche differentiation has been well documented as a mechanism allowing coexistence between sympatric carnivores, for which responses to competition have been attributed to their prey size spectrum [10–12], habitat preferences [13–15] and daily activity rhythms [6,16–18]. Competition between carnivores and their spatial distribution may be determined by not only predation on non-carnivore prey, but also the perceived or real risk of intraguild killing. Much evidence is available on interspecific killing involving different pairs of coexisting carnivore species [19–22], especially felids, which may have sweeping effects on carnivore community structure. Carnivores’ body size and morphological similarity have a strong influence on interspecific competition and killing, and it is expected that interspecific interactions should be higher when species pairs are closer in size [23].

Carnivore population density scales to prey productivity [24,25], but the high expansion of human activities, conducting to habitat loss, landscape modification, poaching and human-carnivore conflicts, are leading carnivores populations to decline worldwide [26–28]. As a consequence of altered anthropogenic landscapes, reductions in both carnivores and prey abundance may have an impact on carnivores’ mechanisms of resource selection, temporal activity patterns, and space use [6,26,27,29].

Despite the key role of trophic interactions in carnivore species coexistence, understanding how much competition and risk of intraguild killing influence large carnivore assemblages remains a challenge. This is mainly due to the difficulty in obtaining data across broad spatial scales required to study these ecological processes, as well as sufficient records of species that frequently occur at low densities and/or exhibit elusive behavior. Most studies discuss species interactions at local scales (e.g.,[11,15,30]), but how predators change their behavior as they move through heterogeneous landscapes remains largely unexplored. Conducting multi-site comparisons of spatial distributions and activity budgets of co-existing wild cats will be important to improve understanding of mechanisms of coexistence.

In this study, we combine data from long-term camera trap monitoring at eight protected forest sites across the Neotropics. Up to six cat species could be found within each study site [31–33]: jaguar (*Panthera onca*), puma (*Puma concolor*), ocelot (*Leopardus pardalis)*, jaguarundi (*Herpailurus yagouaroundi*), margay (*Leopardus wiedii*) and oncilla (*Leopardus tigrinus*). These species spanning a wide range in bodies sizes, with jaguar and puma being the large predators (31–158 kg and 29–120 kg, respectively) and ocelot, jaguarundi, margay and oncilla figuring as smaller cat species (8–15 kg, 4.5–9 kg, 3–9 kg, and 1.5-3kg, respectively) [13,34–38].

**Fig 1:**
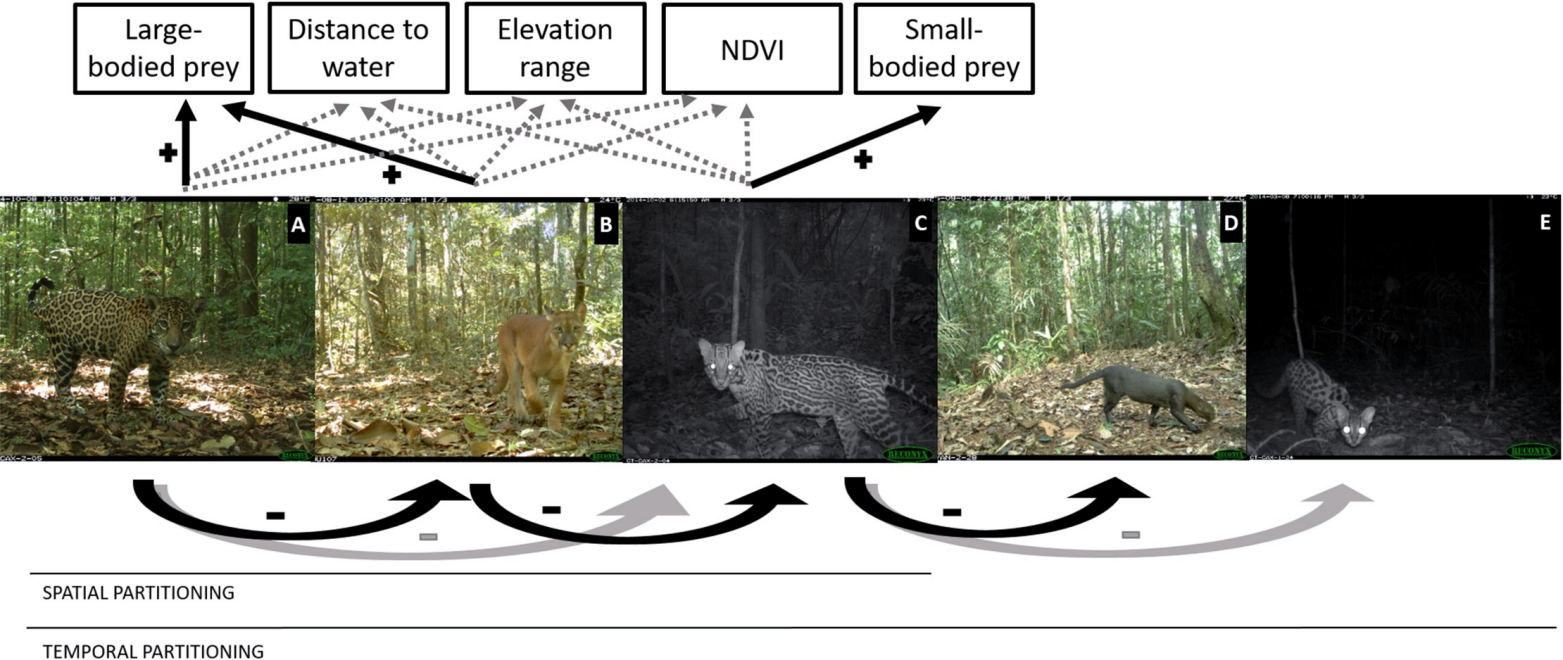
Target Neotropical cat species and summary hypotheses. From large to smaller species: A–Jaguar, B–Puma; C–Ocelot, D–Jaguarundi, and E–Margay. Spatial partitioning hypothesis (including jaguar, puma and ocelot): 1) prey availability would be more important in determining felid habitat use than landscape covariates; 2) based on body weight ratios, jaguar exert negative effects on puma and ocelot, and puma exerts negative effects on ocelot. Temporal partitioning hypothesis (including all five species): higher temporal segregation between species pairs experiencing higher chances of competition. Black arrows indicate strong relationship and grey arrows indicate weaker relationship. Photos by: CAX (A, C and E), COU (B) and YAN (D).

We investigated patterns of niche differentiation between five of the six cat species (excluding oncilla due to limited records) occurring at our Neotropical forest sites (Fig 1), focusing on mechanisms of coexistence at sites under varying levels of integrity. Our study areas are under different landscape contexts (i.e. fragmented or intact forests), and contain different species compositions and abundances of felids and their prey base [31,33]. We used the following approaches: (1) occupancy modelling, as a measure of habitat use, to identify which characteristics (landscape complexity and prey availability) influence habitat use of jaguar, puma and ocelot; (2) occupancy modelling incorporating occupancy estimates of potential competitive cat species to explore spatial co-occurrence among the same three largest Neotropical cats; (3) modelling of temporal activity patterns of the five species (jaguar, puma, ocelot, jaguarundi and margay) to assess and quantify overlaps in temporal activity between felids pairs that are more closely matched in size; (4) modelling of temporal activity patterns within Neotropical cat species to compare temporal activity patterns and activity levels across study sites with differing felid assemblages and potential levels of competition.

Competition and interspecific killing are predicted according to body weight relationships. Food competition should be higher when the larger species was less than twice the size of the smaller one [23], while the intensity of interspecific killing should reach a maximum when the larger species is 2.0–5.4 times as large as the smaller one [21]. Based on that, pumas and jaguars are more likely to compete for food as they have similar body sizes and their distribution are modulated by similar prey [6,11]. The same relationship is expected among ocelot, jaguarundi and margay [6,8]. In addition, jaguars and pumas should exert a strong killing pressure on the ocelot and, in turn, ocelots on the two smaller species, jaguarundi and margay [6].

We hypothesized that there would be spatial segregation among the three largest cats, with large-bodied prey availability being a key factor for jaguar and puma, and small-bodied prey availability for ocelot; but we expected that puma, being a subordinate competitor of jaguar, will vary in its selection of optimal habitat as an avoidance response to jaguar [39]. We therefore hypothesized negative effects of jaguar on puma and ocelot occupancy, negative effects of puma on ocelot, and neutral effects of either puma or ocelot on jaguar occupancy. Regarding temporal interactions, we hypothesized, all else being equal, higher temporal segregation between species pairs experiencing higher chances of competition and intraguild killing (i.e., jaguar-puma and puma-ocelot higher than jaguar-ocelot; and ocelot-jaguarundi and jaguarundi-margay higher than ocelot-margay). Lastly, we are interested if differences within species across sites would be explained by competition pressure, and we hypothesized that there would be temporal shifts, on both activity patterns and activity levels, within the same species between study sites due to low or high occurrence of large predators.

## Methods

### Study sites

**Fig 2:**
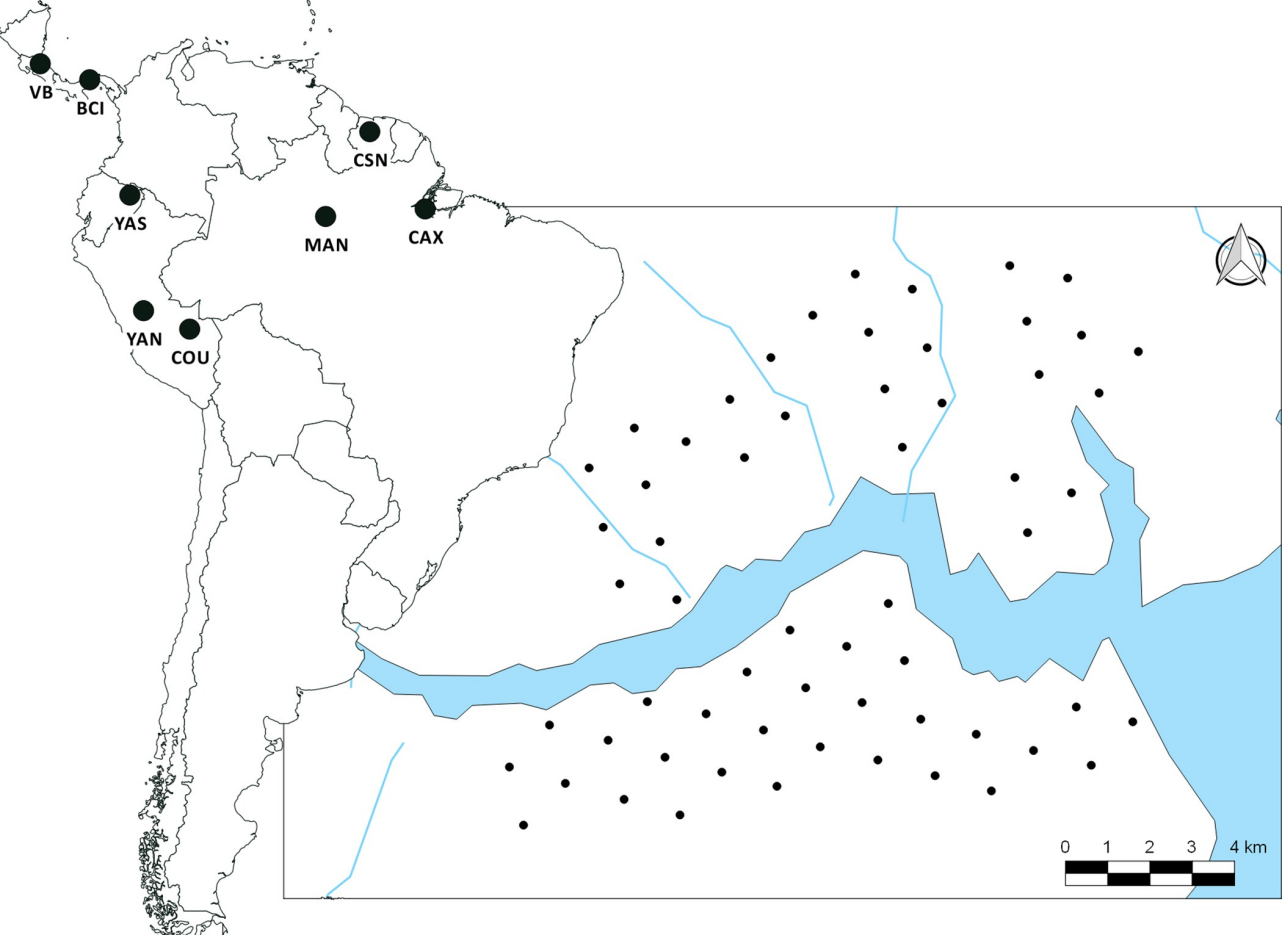
Location of the eight Neotropical study sites and a map of a typical camera trap array at Caxiuanã National Forest (CAX), Brazil. Each point represents a camera trap location. Camera traps are distributed in two sampling arrays of 30 camera traps each (North and South of Caxiuanã River) See site codes on Table 1.

We used data from eight Neotropical forest sites distributed across six countries in Central and South America (Table 1; Fig 2). Data are part of the Tropical Ecology Assessment and Monitoring (TEAM) Network, a global standardized monitoring program for terrestrial vertebrates based on camera-trapping.

**Table 1:** Location and area of the eight Neotropical forest sites analysed in this paper.

Code	Study site, Country	Longitude, Latitude	Area (ha)	Landscape type^a^
BCI	Barro Colorado Nature Monument, Panama	-79.851, 9.092	32631.22	FR
CAX	Caxiuanã National Forest, Brazil	-51.534, -1.775	471192.63	CF
COU	Cocha Cashu—Manu National Park, Peru	-71.409, -11.843	1704505.53	CF
CSN	Central Suriname Nature Reserve, Suriname	-56.207, 4.741	1630233.61	CF
MAN	Manaus, Brazil	-59.935, -2.415	1198944.01	FR
VB	Volcan Barva Transect, Costa Rica	-84.021, 10.422	49502.04	FR
YAN	Yanachaga National Park, Peru	-75.303, -10.316	293234.07	FR
YAS	Yasuni Research Station, Ecuador	-76.458, -0.609	1040686.74	CF

^a^Classification based on [33]: FR–fragmented forest and CF–continuous forest.

Our study sites consist of intact protected forest landscapes, in which formal protected areas were either indistinguishable from the continuous forest in surrounding areas (i.e., CAX, COU, CSN, and YAS) or fragmented forest landscapes in which protected areas were embedded within a patchwork mosaic of forest and non-forest areas (i.e., BCI, MAN, VB, and YAN) (See categorization criteria for landscapes in [33]).

### Data collection

We collected data on five Neotropical cats, *Panthera onca* (jaguar), *Puma concolor* (puma), *Leopardus pardalis* (ocelot), *Herpailurus yagouaroundi* (jaguarundi), and *Leopardus wiedii* (margay), following the standardized TEAM protocol for monitoring terrestrial vertebrates [40,41]. The sampling design consisted of a set of regular grids of 60 camera trap stations at a density of one camera per 2 km^2^ (spaced approx. 1.4 km apart), corresponding to a sampling area of ≈ 120 km^2^ at each site (Fig 2). Camera traps were deployed once a year at the same camera trap station, remaining in the field for at least 30 days (ranged 30–60 days) during the dry season at each site (or months with <200 mm mean rainfall). Each year of survey (i.e, 60 camera traps X 30 days) was defined as a sampling period.

The total number of sampling period varies between study sites (2–10 years of data), because monitoring protocol was implemented in different moments at each site. Therefore for occupancy modeling, we performed exploratory analysis to select the ideal time interval to group the data for analysis, and then used five sampling periods at each study site (except for Manaus where only two surveys were available) (Table 2, See Data Analysis for details). Camera traps (Models RM45 and HC500, Reconyx Inc.) were setup to take three pictures per trigger with no delay between photos, working 24 hours/day. No baits were used to attract animals (Detailed information about the implementation protocol is available on [40,41]).

**Table 2:** Sampling period analysed, sampling effort, number of detections (Detc), records per 100 CT/days (RAI), and estimated occupancy probability^1^ (ψ) from single-season models of the Neotropical cats’ species in eight protected forest sites.

Site	Number of Sampling periods (years)	Effort	Jaguar			Puma			Ocelot			Jaguarundi			Margay			Oncilla		
			Detc	RAI	Ψ (±SE)	Detc	RAI	Ψ (±SE)	Detc	RAI	Ψ (±SE)	Detc	RAI	Ψ	Detc	RAI	ψ	Detc	RAI	ψ
BCI	5 (2010–2014)	9199	0	-	-	0	-	-	196	2.13	0.74 (0.17)	10	0.11	-	4	0.04	-	0	-	-
CAX	5(2010–2015)	11395	36	0.32	0.45 (0.14)	58	0.51	0.43 (0.03)	43	0.38	0.57 (0.09)	2	0.02	-	28	0.25	-	0	-	-
COU	5 (2011–2015)	9481	46	0.49	0.50 (0.16)	45	0.47	0.46 (0.03)	283	2.98	0.67 (0.14)	9	0.09	-	9	0.09	-	6	0.06	-
CSN	5 (2008–2012)	11107	39	0.35	0.47 (0.16)	39	0.35	0.43 (0.04)	127	1.14	0.60 (0.10)	22	0.20	-	33	0.30	-	2	0.02	-
MAN	2 (2010–2011)	4600	10	0.22	0.34 (0.09)	5	0.11	0.37 (0.06)	18	0.39	0.63 (0.09)	4	0.09	-	0	0	-	0	-	-
VB	5 (2012–2016)	6971	2	0.03	-	37	0.53	-	21	0.30	0.62 (0.11)	0	-	-	1	0.01	-	1	0.01	-
YAN	5 (2011–2015)	8249	23	0.28	0.37 (0.08)	17	0.21	0.47 (0.03)	60	0.73	0.60 (0.09)	16	0.19	-	7	0.08	-	0	-	-
YAS	5 (2012–2016)	11833	30	0.25	0.62 (0.18)	54	0.46	0.43 (0.04)	167	1.41	0.66 (0.12)	18	0.15	-	17	0.14	-	0	-	-

^1^Occupancy probability and standard deviation estimated by model averaging.

In addition to the target species’ data, we collected information on prey species of jaguar, puma, and ocelot using the images from the camera traps. The use of camera trap images to assess prey availability has been adopted in previous carnivore studies, for both occupancy [8,42,43] and detection probabilities [44]. This was possible because the method allows recording a wide range of ground-dwelling mammals and birds, most of them medium to large-sized species. Data from mammals and birds with body size < 1Kg, recognized as jaguarundis and margays’ prey [8,10,34], were not recorded because their occurrence would be probably under-represented given the method used [40].

### Ethics statement

Field activities have been developed in partnership with each protected area site. During this research, the animals were photographed through camera traps in their natural environment and none of them were captured, handled or sacrificed. Therefore, there are no protocols to be reported to institutional or governmental agencies that regulate animal research.

### Covariates

For each camera trap station we recorded variables associated with landscape complexity (elevation range, distance to the nearest water source, slope and the Normalized Difference Vegetation Index—NDVI), food resources (prey availability) and species interactions (occupancy estimates of potentially competing cat species). Elevation and slope data were calculated using a digital elevation model (DEM) based on the NASA Shuttle Radar Topographic Mission (SRTM), with spatial resolution of one arc-second (≈ 30m). Elevation range was obtained by the difference between the higher and lower elevation of camera traps station within each study site. Normalized Difference of Vegetation Index (NDVI), was generated from eMODIS NDVI scenes (Vegetation monitoring). We obtained the mean NDVI at a buffer of a 500 meters radius around each camera trap point. Data of DEM and eMODIS were downloaded from the U.S. Geological Survey [45] and the estimates were made using QGIS software [46]. Distance to the nearest water source (river/streams) was estimated using hydrological shapefiles from HydroSHEDS [47] in QGIS software [46] and the R package *Fossil* [48].

Prey availability at each camera trap station was inferred using the camera data of potential prey species (ground-dwelling mammals and birds; See S1 Table for prey species list at each site). Firstly, prey images were separated assuming a 1-hour interval between consecutive photos to ensure the records were independent [49,50]. Prey availability was defined as the ratio between the total number of prey records and the sampling effort for each camera trap station in each sampling period [51–53]. We subdivided prey species into two categories [44]: 1) Large-bodied prey: mammals and birds with a body mass greater than 15 kg, and 2) Small-bodied prey, mammals and birds with a body mass less than 15 kg. These categories are based on dietary preferences of jaguar and puma (which mostly consume medium to large-bodied prey [54,55]), and ocelot (which consume small to medium-bodied prey [10,18]). Prey body mass data were obtained from the EltonTraits1.0 database that includes information on key descriptors of the foraging ecology of birds and mammals [56]. We normalized all covariates and used Spearman’s rank correlations to test for collinearity. Only covariates with low correlation (ρ > 0.70) were used (S2 Table).

### Data analysis

#### Spatial partitioning

We used single-species occupancy models with a likelihood-based approach to estimate the occupancy (ψ) of jaguar, puma and ocelot, and assess habitat use and intraguild interactions, while accounting for detection probability [57,58]. Because data for jaguarundi, margay, and oncilla were restrict to few records in most of the study sites and/or species were not recorded during consecutive sampling periods (preventing species pairs comparisons), we did not perform occupancy analysis for these three smaller cats (Information about detections and relative abundance were given at Table 2).

We organized the detection histories of each species by dividing each of the sampling periods into sampling occasions of five days each [53]. We adopted a single-season analytical approach, wherein data from five sampling periods at each study site were stacked, as independent surveys in modelling procedures. Single-season modelling was chosen because our data were too sparse to fit multi-season occupancy models, which estimates additional parameters (colonization/extinction). Also, this was based on the assumption that annual variation in detection probability and occupancy (and the relationship between occurrence and habitat covariates) would be minimal over the time-frame of the study. We therefore developed models to formally assess the effect of time (multiple sampling periods) in occupancy and detection. We allowed psi (ψ) to be constant and to vary according to study site and time (i.e., sampling period) or a combination of both, and then we assumed the same for detection probability (*p*) using all possible combinations between parameters and covariates. Model selection results provided no evidence that time had a marked influence on occupancy and detection probabilities (S3 Table). From this, we relaxed the basic occupancy modelling assumption that sites are closed to population changes [58,59] and broadly interpreted occupancy as a measure of local habitat use, instead to “true occupancy”, considering that the presence of a species at a camera trap station occurs completely by chance [57].

We used a multi-stage approach while modelling the occupancy of each cat species (similar to [9,60]). We first built models to find the main covariates influencing for detection probability prior to performing model selection to investigating habitat use [57]. We constrained occupancy to be constant (ψ (.)) and allowed p to vary by a single covariate or a combination of covariates (additive effects) [57]. The covariates used in detection (p) models reflect habitat characteristics and/or access to resources that likely to affect animal behavior and, consequently, species’ detection. We also introduced a categorical variable, referring to “study site”, which account for factors that can influence detection due to slightly different field procedures and local habitat characteristics. Covariates for p were: elevation range, NDVI, large-bodied prey availability for jaguar and puma models, and small-bodied prey availability for ocelot models, and study site.

For the next stage, we developed a second model set to determine the most influential habitat factors for occupancy. We allowed ψ to vary by a single covariate or a combination of two covariates, and fixed detection covariate(s) to those selected from the previous step for each species. We selected covariates for occupancy models that may reflect habitat preferences: elevation range, distance to the nearest water source, NDVI, availability of small-bodied prey and large-bodied prey. We hypothesized that prey availability would have a positive effect on habitat use, with large-bodied prey being a key factor for jaguar and puma, and small-bodied prey for ocelot. We were interested in the possible difference between the two most similar species (jaguar and puma), so we expected that puma will vary in its selection of optimal habitat.

Finally, in a third step we used single-species occupancy models to examine species co-occurrence by including occupancy estimates of jaguar, puma and ocelot from previous step as a potential covariate in predicting occupancy. By assuming that the influence of larger-bodied species is more intense on smaller ones, either by interference competition or interspecific killing [6,19,23], we built models to examine if habitat use is significantly influenced by the occurrence of a reciprocal competitor. In this way, for example, if jaguar are significantly influencing the spatial distribution (and hence habitat use) of puma or ocelot, then we would expect a significant association in the model. We therefore hypothesized negative effects of jaguar on puma and ocelot, negative effects of puma on ocelot, and neutral effects of either puma or ocelot on jaguar occupancy. We evaluated species interactions models including the most supported habitat models (ΔAIC < 2 from step 2) in the model set for each species, and comparing AIC values and models weights [9,61].

We assessed candidate models and estimated parameters for each modelling step using the R package *Unmarked* [62,63]. We performed a multi-model selection procedure based in Akaike's Information Criterion (AIC) and model fits were evaluated using the overdispersion parameter (ĉ) on the saturated model (including all covariates, e.g., ψ (small+large+elevation+dist.water+ndvi)) by running a goodness-of-fit test [57,61]. Models with ΔAIC < 2 were considered to have substantial support and ĉ was used to correct AIC for overdispersion (QAIC) [61]. When several models obtained AIC support, we applied model averaging to obtain occupancy and detection estimates, using the R package *AICcmodavg* [61,64].

Additionally, we assessed the relative importance of each covariate by summing the Akaike weights (AIC_wt_/QAIC_wt_) of all the models in which that covariate was present [61]. When models set do not contain the same number of each covariate, we divided the cumulative model weights for a particular variable by the number of models containing that variable to get an average weight (AIC_wt_/QAIC_wt_) [61]. We used beta coefficients to determine whether the influence of a covariate was negative or positive and calculated the 95% confidence intervals for the model averaged estimates to discriminate the importance of individual variables [57,61,64]. When 95% CIs of beta estimates did not include 0, we concluded that the given covariate has a strong effect on habitat use [61].

#### Temporal partitioning

We used time and date recorded in the images of all camera traps and surveys to describe daily activity patterns, activity levels and temporal overlap. Analyses were performed when species presented a minimum of ten images at each study site [65]. Time of day was converted to solar time (i.e., adjusted according to sunrise and sunset) and anchored in the equinoctial algorithm (pi/2 and pi*3/2) for all study sites, allowing the comparison between different time zones [66,67], using the *Insol* package in R [66].

Activity pattern (i.e., distribution of activity of an animal throughout the day) was estimated using the Kernel circular density function [68,69]. To quantify overlap between daily activities we used the overlap coefficient (Δ), which varies from 0 (no overlap) to 1 (total overlap). We used Δ1 and Δ4 estimators when the number of images was <75 and ≥75, respectively [69,70]. Confidence intervals were obtained from 999 smoothed bootstrap samples. Analyses were conducted using the *Overlap* and *Activity* packages in R [70,71]. As the overlap coefficient is a descriptive method, we compared the activity patterns of each species pairs using Watson's two-sample test (U^2^) in the *Circular* package, which is a homogeneity test for circular data, where values for U^2^ inform if two samples belong to the same parent population (H_0_) or differ significantly [65,72]. Based on morphometric similarity and greater probability of competition and intraguild killing [23], we hypothesized higher temporal segregation between species pairs experiencing higher chances of competition.

Finally, we investigated whether activity patterns and activity levels (i.e., proportion of hours/day that an animal is active) within the same species across study sites can be explained by competitive pressure. We expected temporal shifts within the same species between study sites due to low or high occupancy of large predators. We then assumed that the pressure of competition and/or killing risk would be determined by a ranking based on occupancy estimates of jaguar, puma and ocelot from previous spatial analyses (and camera trap rates for jaguarundi and margay). Intra- and inter-specific comparisons of activity levels were implemented using a Wald test in the *Activity* package [71].

## Results

Five years of camera-trapping at each of the eight study sites amounted to a total sampling effort of 72,835 camera trap days across 480 camera trap stations, yielding 186 records of jaguar (*Panthera onca*), 255 of puma (*Puma concolor*), 915 of ocelot (*Leopardus pardalis*), 81 of jaguarundi (*Herpailurus yagouaroundi*), 99 of margay (*Leopardus wiedii*) and nine of oncilla (*Leopardus tigrinus*) (Table 2).

### Spatial partitioning

#### Detection probability

Two ‘best’ models supported large-bodied prey, study site and elevation as the main predictors for jaguar detection probability, while highest-ranking models indicated large-bodied prey and elevation as important in explaining puma detectability (S4 Table). For ocelot, the two top-ranked models for detectability included all possible predictors. On the basis of AIC/QAIC and model weights, we selected the most parsimonious model of each species while running occupancy models, capturing the main features of the data [57] (S4 Table).

#### Occupancy probability

**Fig 3:**
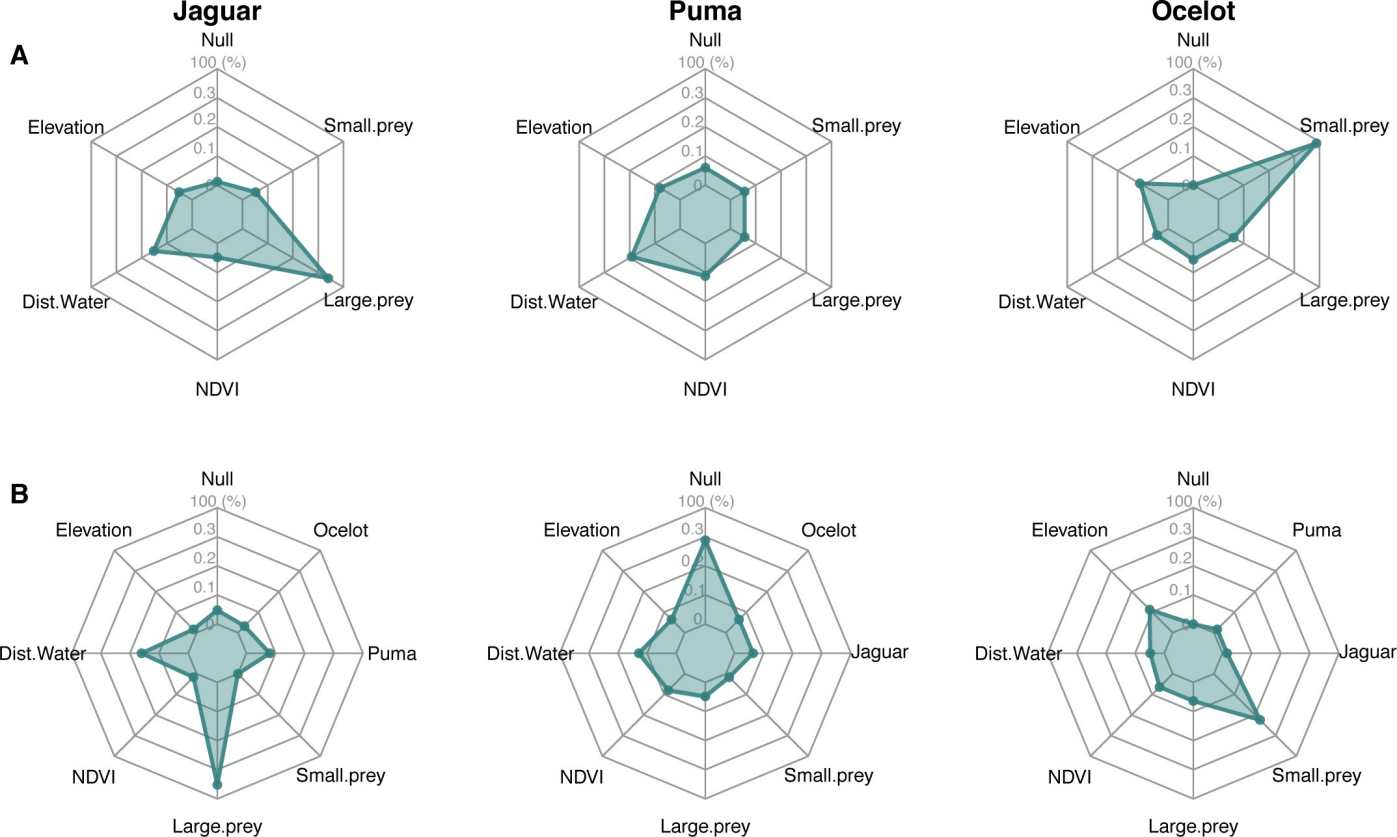
Relative importance of environmental and interaction covariates on the habitat use of three Neotropical forest cats. Row A–Sum of models weights (AIC_wt_/QAIC_wt_) of occupancy models to assess habitat factors; row B–Sum of models weights (AIC_wt_/QAIC_wt_) of occupancy models to assess both habitat factors and species interactions.

**Fig 4:**
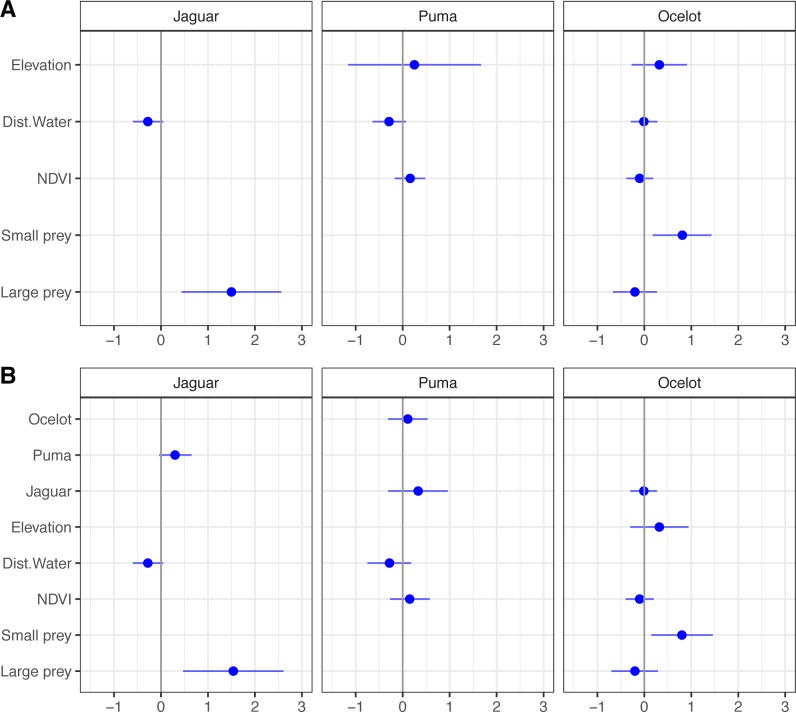
Covariates effect on habitat use of jaguar, puma and ocelot. Beta estimates with 95% of confidence interval estimated from single-season species models: row A—Beta estimates from occupancy models to assess habitat factors; row B–Beta estimates from occupancy models to assess both habitat factors and species interactions (The beta estimates has an effect on the dependent variable when confidence interval do not include 0).

Two occupancy models were supported for jaguar in habitat models set (AIC < 2), with a significant positive effect of large-bodied prey availability (Figs 3A and 4A; S5 Table). As expected, adding puma and ocelot occupancy estimates had no influence on jaguars’ habitat use (Figs 3B and 4B; S6 Table). Even with the covariate ‘puma occupancy’ being first-ranked in the models set accounting for species interactions, only large-bodied prey strongly affected jaguars’ habitat use (based on 95% IC; Fig 4B and S6 Table).

For puma, five models received support, but none of the best-ranked covariates (distance to the nearest water source, NDVI, and elevation) represented a strong effect on habitat use (Figs 3A and 4A; S5 Table). Adding jaguar and ocelot occupancy estimates improved the fit of puma models incorporating habitat covariates (Fig 3B), but both species had no significant influence on pumas’ habitat use (Fig 4B and S6 Table).

Five models had substantial support for ocelot occupancy with a positive effect of small prey availability emerging as the most important predictor. Elevation, large-bodied prey, NDVI and distance to nearest water source were also ranked highly, but only small-bodied prey had a significant effect (Figs 3A and 4A; S5 Table). Model set accounting for species interactions also supported five models, but only small-bodied prey had a large effect on ocelot occupancy, contradicting our hypothesis (Figs 3B and 4B; S6 Table).

### Temporal partitioning

**Fig 5:**
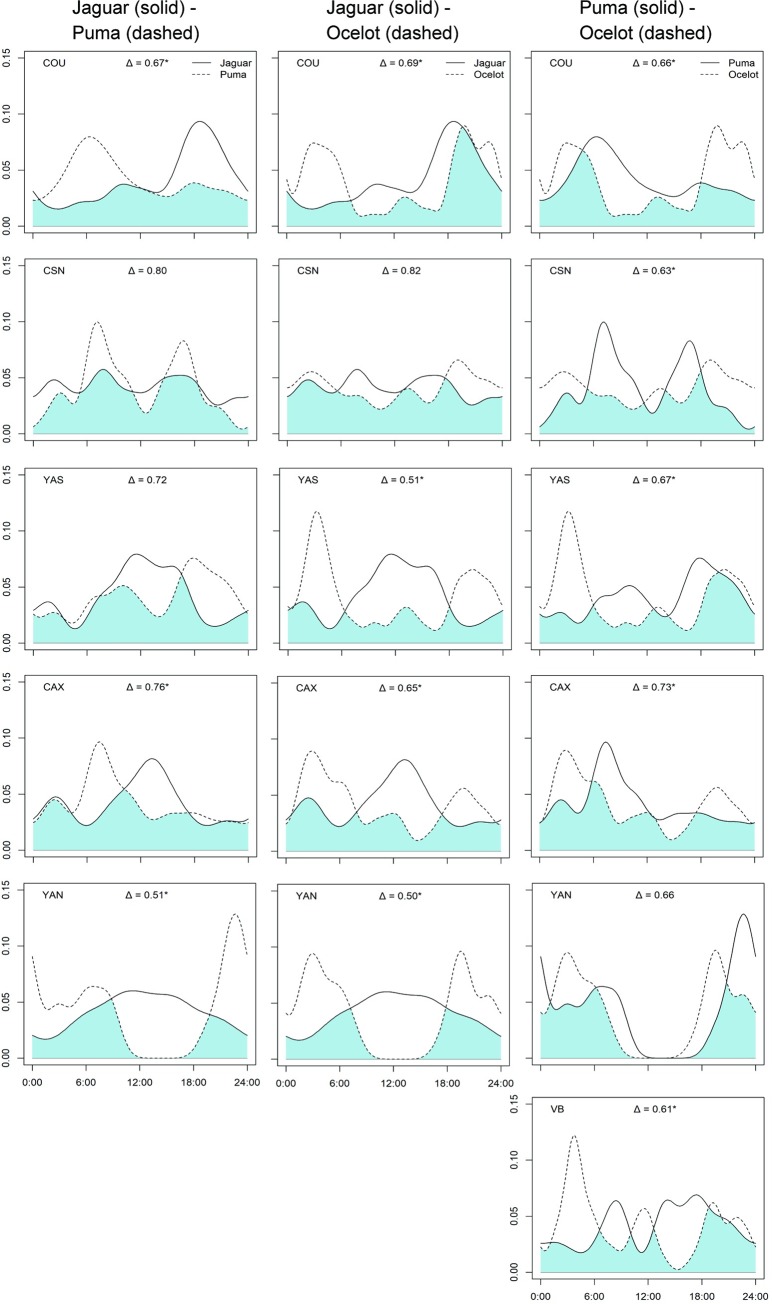
Coefficient of overlap in daily activity patterns between jaguar, puma and ocelot in Neotropical forest sites. X and Y axis represent time of the day and activity density, respectively. Overlap is represented by blue shaded areas and Δ is the coefficient of overlap (varying from 0 –no overlap to 1 –total overlap). (*) indicates significant differences. Study site is indicated in the top left corner.

**Fig 6:**
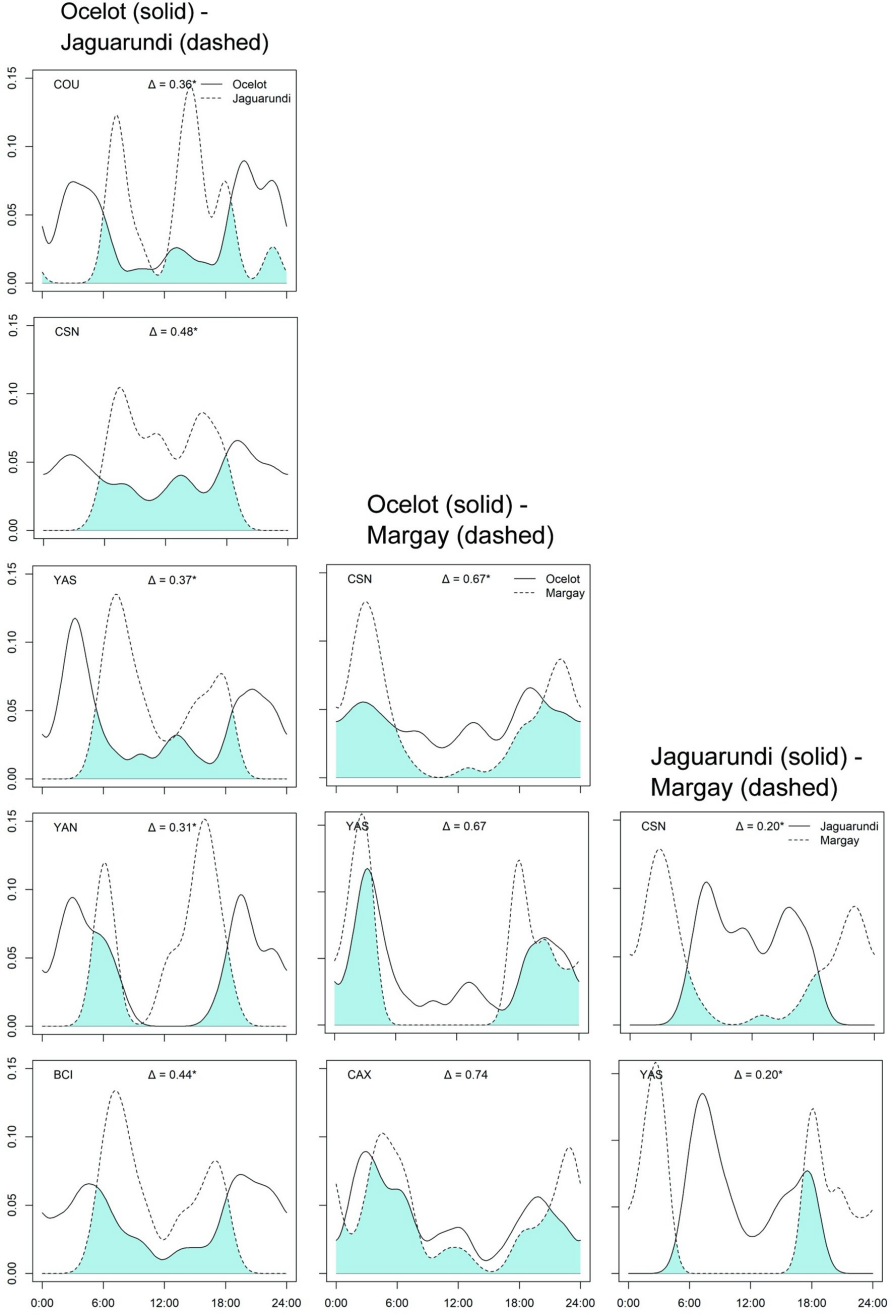
Coefficient of overlap in daily activity patterns between ocelot, jaguarundi and margay in Neotropical forest sites. X and Y axis represent time of the day and activity density, respectively. Overlap is represented by blue shaded areas and Δ is the coefficient of overlap (varying from 0 –no overlap to 1 –total overlap). (*) indicates significant differences. Study site is indicated in the top left corner.

There was a moderate degree of temporal overlap between jaguar, puma and ocelot activity patterns, with the peaks of activity differing between most of the analyzed species pairs (Fig 5; S7 Table). We observed an overlap average of Δ = 0.69 for jaguar-puma, Δ = 0.63 for jaguar-ocelot, and Δ = 0.66 for puma-ocelot. Higher coefficients of overlap were observed for jaguar-puma and jaguar-ocelot pairs at CSN (Δ > 0.79) and lower overlap was observed for jaguar-puma at YAN (Δ = 0.50) and jaguar-ocelot at YAN and YAS (Δ < 0.50). Considering the smaller cats, pairwise activity overlap in ocelot-jaguarundi were low for all sites (average of Δ = 0.39), while ocelot-margay on average overlapped by Δ = 0.69. Jaguarundis and margays could only be compared across two sites, but showed the lowest activity overlap (mean Δ = 0.20), due to their nearly opposite temporal activity (Fig 6; S7 Table). Low numbers of jaguarundi and margay photographic detections prohibited detailed analysis of overlap activity for all study sites, but we observed from other 6 records of jaguarundi (CAX: 2; MAN: 4) and 21 records of margay (BCI: 4; COU: 9; VB: 1; YAN: 7) that species were active in the same time period observed during overlap analysis described above (Fig 6), with jaguarundi active during daylight and margay being more active during night time.

**Fig 7:**
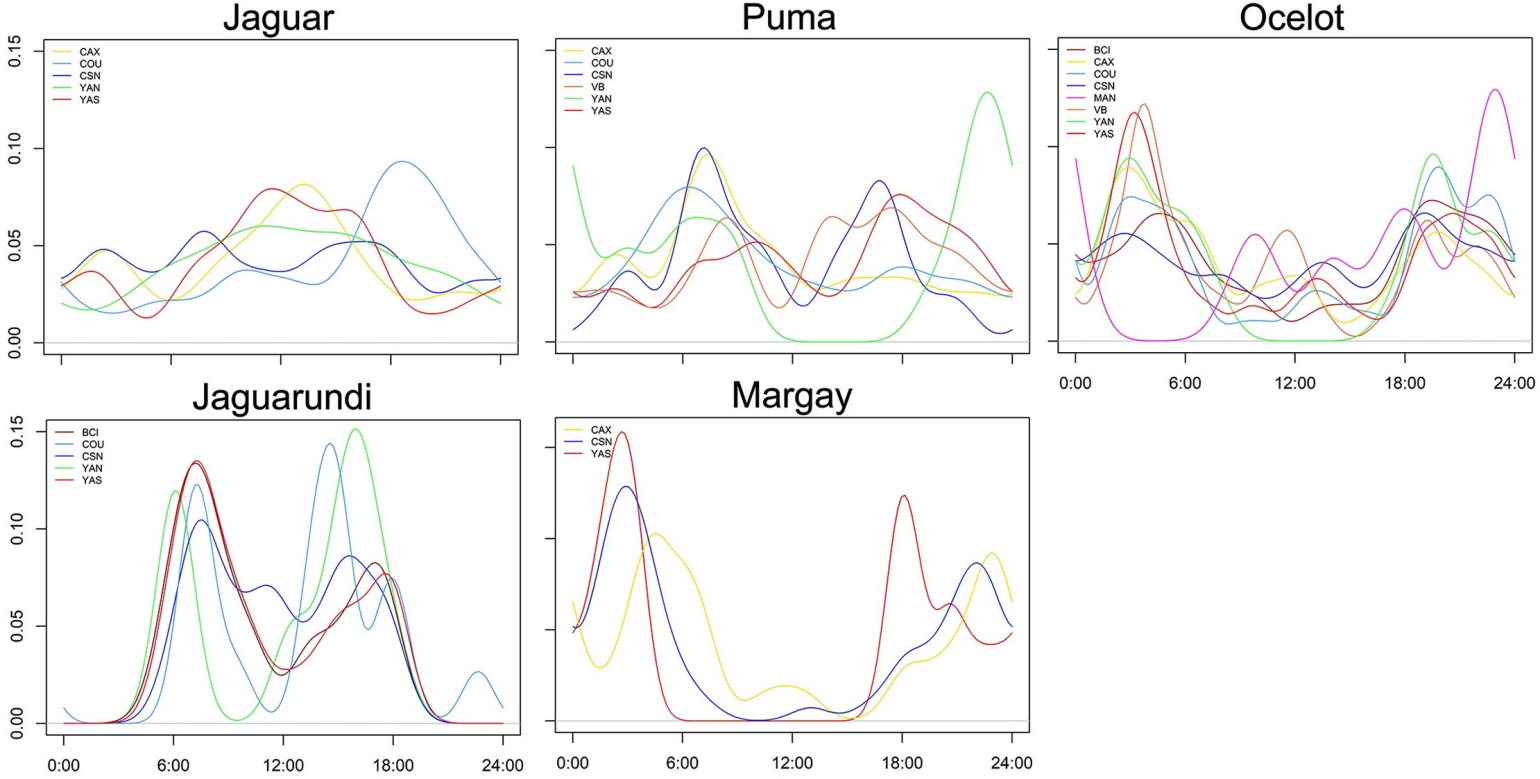
Intraspecific variation in daily activity patterns in felid species across eight Neotropical forest sites. X and Y axis represent time of the day and activity density, respectively.

Examining temporal shifts within the same species across study sites, we observed that jaguars were mainly active during the day at CAX, YAN and YAS sites (>60% of activity between 06:00h and 18:00h), but exhibited a cathemeral activity pattern at CSN. Nevertheless, differences were only significant when these sites were compared with COU, where jaguar exhibited a nocturnal peak (40% of activity between 18:00h – 00:00h) (Fig 7; S8 Table). Puma showed a non-uniform pattern, showing different activity peaks across sites (Fig 7). Overlap within puma populations was on average Δ = 0.71 (range = 0.50–0.88) and activity pattern differed significantly (S8 Table).

Ocelots were mainly active during crepuscular and nocturnal periods (>60% of activity between 18:00h and 06:00h). The only exception was CSN, where ocelots showed a cathemeral pattern. Temporal overlap within ocelots across sites was high (mean Δ = 0.79; range = 0.67–0.88). Jaguarundis exhibited a completely diurnal pattern across all sites with a bimodal activity peaks around dawn and dusk, while margays were strictly nocturnal (~70% of activity between 18:00h and 06:00h). Both species showed no significant differences in their activity period across sites (Fig 7; S8 Table).

**Fig 8:**
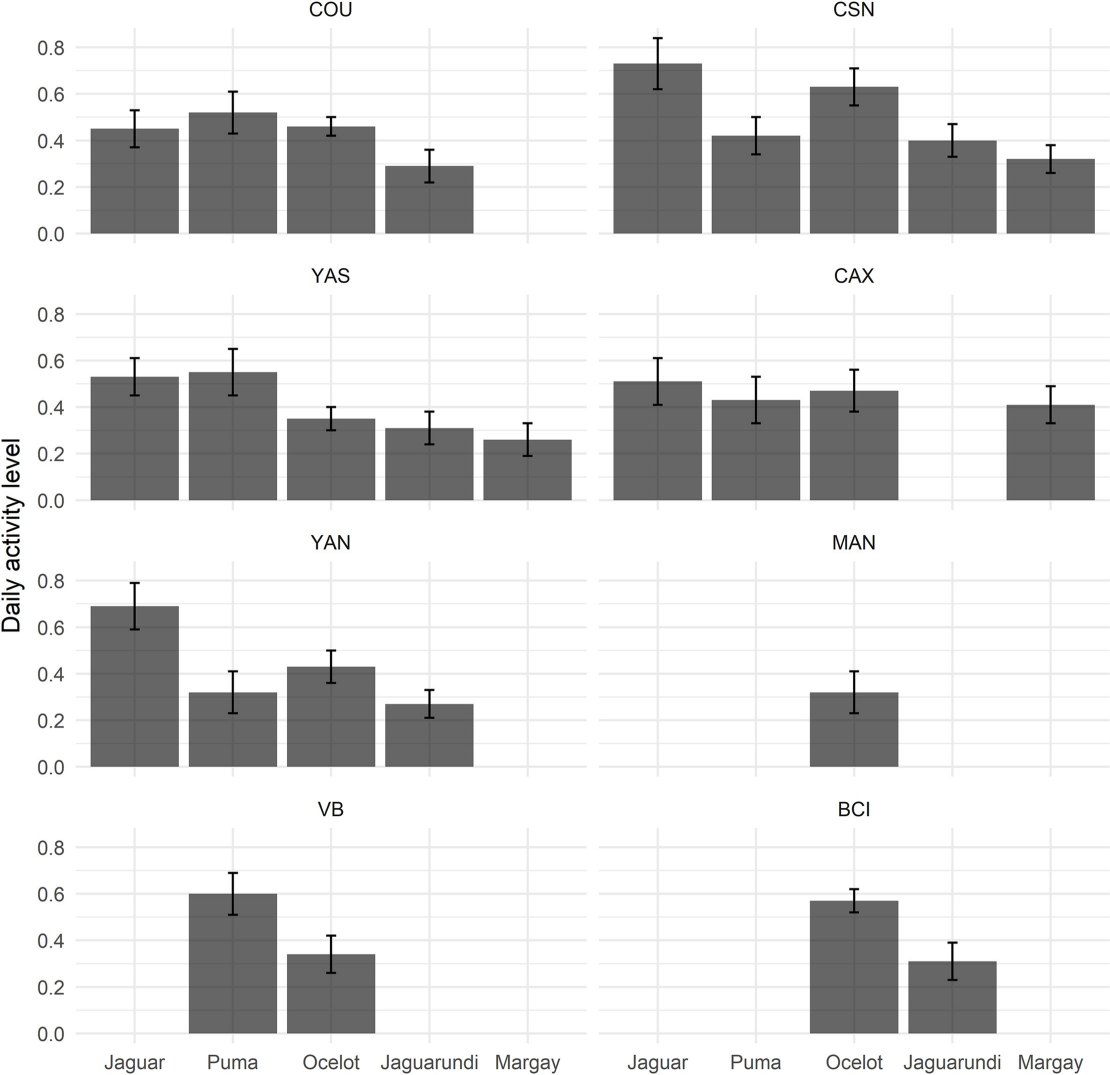
Daily activity level of felid species across the eight Neotropical forest sites. Proportion of active hours per day. Error bars represent the standard error.

The overall activity levels (proportion of time spent active) were 0.58 (SE = 0.09) for jaguar, 0.47 (SE = 0.09) for puma, 0.45 (SE = 0.07) for ocelot, 0.32 (SE = 0.07) for jaguarundi and 0.33 (SE = 0.07) for margay (Fig 8). Considering the effect of predator pressure, activity level of puma was higher at VB, where jaguar had the lowest abundance. However, differences are statistically significant only between VB and YAN sites (Wald χ^2^ = 4.67, df = 1, *p* = 0.03; S9 Table).

Ocelot daily activity levels were higher at CSN, which differed significantly to other sites (except when compared with CAX and BCI). Ocelot activity level was also higher at BCI, where large-bodied cats are missing and ocelots are essentially the top-predator. Differences were statistically significant between BCI and three other sites: MAN (Wald χ^2^ = 5.66, df = 1, p = 0.01), VB (Wald χ^2^ = 6.30, df = 1, p = 0.01) and YAS (Wald χ^2^ = 10.03, df = 1, p *<* 0.01).

Jaguarundis and margays were active for a similar proportion of time, regardless of ocelot occupancy patterns. Margay activity was higher at CAX, where ocelot occupancy was lower, but differences were not significant (Fig 8; S9 Table).

## Discussion

Our study explored how environmental and species interactions affect the habitat use and activity patterns of forest felid assemblages in the New World tropics. The patterns and assemblage structure observed at our eight study sites are congruent with previous studies in Neotropical forests [6,29,31], with the two large-bodied cats consistently showing their highest abundances in large tracts of protected forests, the ocelots being numerically dominant at most of the sites, regardless of their conservation status and forest extent, and the smaller cats appearing as less abundant species.

### Species habitat use and spatial partitioning

Occupancy models accounting for detection probability showed evidence that niche differentiation between jaguar, puma, and ocelot according to prey preferences is a potential mechanism of coexistence. Jaguars and ocelots occupancy was closely related to prey availability [24,55], which helps explain differences across sites. Large-bodied prey were more abundant at sites where jaguar occupancy estimates were higher (e.g. YAS and COU). Conversely, low incidence of large-bodied prey abundance matched low rates of jaguar occupancy (e.g. MAN and YAN). At YAN site, for example, two important ungulate prey species of jaguar—brown brocket deer (*Mazama nemorivaga*) and white-lipped peccary (*Tayassu pecari*)—failed to be recorded during the entire camera trapping monitoring.

Even though models reflected some well-known relationships, like jaguars and pumas presenting positive associations with water bodies [14,73], only prey availability emerged as an important covariate in determining jaguars’ space use. Puma was not significantly influenced by any covariates. Also, models evaluating species interactions showed no evidence of avoidance of puma to the jaguar, and vice-versa. These findings agree with other studies that shown no spatial segregation between jaguars and pumas [44,74], and potentially species may adopt other mechanisms to allow coexistence, as the use of different food resources and/or partitioning of their activity period [6,67,75].

Our hypothesis that smaller-bodied predators behaviorally evade larger apex predators was framed based on the notion that the local distribution of a top predator may be shaped by resource availability, while the distribution of a mesopredator is largely related to predation risk [76]. Although we did not find a significant influence of jaguar on the spatial distribution of puma, our results suggest that jaguar selects habitats based on high prey abundance, whereas puma display sufficient plasticity in habitat use, indicated by the lack of significance for any covariates in the top-ranked models, and this probably reduces convergence in the use of similar resources with jaguar [15,77]. Pumas are considered to be more opportunistic predators, being observed at fragmented and human-modified forest landscapes, which have a heavier impact on jaguars [15,73,77].

Regarding ocelots, its distributions were strongly influenced by small-bodied prey rather than other habitat covariates or by occupancy estimates of the two largest predators. These findings supported the idea that ocelot does not meaningfully compete for food resources with either jaguar or puma [18]. Competition between puma and ocelot is expected to be higher when jaguar is relatively abundant [78], but competitive exclusion between these species is at best unlikely given the lack of interaction we observed. Our results agree with other studies showing spatial co-occurrence between pumas and ocelots [6,74], and observing that detection probability can be higher when the other species were present in the same camera trap station [6]. Furthermore, ocelot occupancy was high both at sites where the two largest-bodied felids were either absent (BCI) or rare (YAN and MAN), but also at sites where these apex predators were relatively common (YAS and COU).

Our results are according with studies involving other sympatric mammalian carnivores. In general, species with similar ecological requirements were often more likely to overlap spatially [7], and habitat features were more important in maintaining the distribution and structure of carnivore guild than species interactions [9].

### Temporal partitioning

Another coexistence mechanism explored in our study was temporal partitioning [6,79]. In support of our temporal segregation hypothesis, we observed that the activity patterns of species pairs (i.e. jaguar-puma, jaguar-ocelot and puma-ocelot) overlapped to a moderate degree, and were significantly different in pairwise comparisons of activity at most sites. Because of the greater morphological similarity between jaguar and puma, we expected a lower degree of overlap between them compared to jaguar-ocelot and puma-ocelot pairs, but this was not confirmed. However, other studies observed that top predators exhibit similar daily activity cycles [44,80,81], indicating that some degree of temporal overlap would be expected from the similar dietary profiles of jaguars and pumas. It is more likely that the general temporal patterns can be related to the attractiveness of food resources, rather than avoidance of a larger predator [11,67].

Jaguar and puma are able to adjust their activity to reduce their foraging energy expenditure, by matching their activity to that of their main prey species [30,67]. We cannot rule out the option that prey abundance and some other habitat characteristics affects temporal activity [29], and consequently temporal partitioning between apex predators. Indeed, the lowest overlap between jaguar and puma was observed at the Ecuadorian site (YAN, Δ = 0.51). Perhaps this is likely associated with the absence of some species and low abundance of large-bodied prey, as stated above. Further analysis considering more detailed habitat characteristics and human disturbance factors are required to understand the relationship between the daily activities of predators facing differences in prey availability.

Considering the smaller felids, our hypotheses of low overlap in activity patterns were confirmed for both the ocelot-jaguarundi and jaguarundi-margay species pairs, which are closest in terms of body weights [34]. This is consistent with a study in the Brazilian Atlantic Forest [6], which suggested that jaguarundis reduce interference competition with the larger ocelots, and avoid competition with similarly-sized margays, by selecting opposite time-periods for their activities. Also, even with ocelots and margays overlapping in their activity patterns, some adaptations for an arboreal life permit the margays to explore a different niche from ocelots [34,36]. A study in Atlantic forest remnant using co-occurrence analysis found no evidence that ocelot have a negative influence on how the margay use the habitat [8].

A final approach in our activity pattern analysis was to investigate if competitive pressure, here measured as occupancy of larger-bodied predators (or abundance for jaguarundi *vs* margay), could explain shifts in activity patterns and levels across study sites. Daily activity patterns within pumas across sites reinforced the notion that temporal shifts in jaguar activities have an impact on sympatric pumas, which tends to concentrate its activities away from the peak of jaguar activity. Moreover, when jaguars are virtually absent, as in VB, pumas extended their activity, with diurnal peaks between mid-day and dusk. Similar results were observed in daily activities patterns of puma and leopard in areas with high or low abundances of apex predators (jaguar and tiger, respectively), likely as an evasive response in side-stepping direct encounters when dominant species are most active [29,82].

Ocelots showed temporal segregation in relation to jaguars and pumas, but temporal activities were unlikely modulated by intraguild killing pressure, and nocturnal activity was also observed across several Neotropical landscapes [6,12,83–85]. Degree of overlap between ocelot populations did not support our hypothesis of competitive pressure, and ocelots were active during similar periods of the day at BCI, where large cats are absent, VB and MAN, where detection rates were low, and YAS and COU, where large cats were far more common.

Jaguar was active for the same proportion of time in most of the sites, as well as pumas, jaguarundis and margays, and differences in activity across sites were mostly not significant. These felid species were therefore active during similar amounts of time regardless of the occurrence of larger predators. Despite significant differences in activity levels of ocelots, no clear pattern could be identified across sites with either higher or lower occurrence of top predators. Due to the large effect found between ocelots’ habitat use and small-bodied prey in our occupancy analysis, we expect that further studies evaluating factors other than competition pressure of a larger predator may explain differences on activity levels.

## Conclusions

This is the first study providing a large-scale insights into the co-occurrence of five forest hyper-carnivore species throughout the Neotropical region, assessing patterns across protected areas of differing size and intactness. We have shown that jaguar, puma and ocelot exhibit clear spatial preferences at local to landscape scales according to prey availability. We found that prey availability is more important for felid space-use than either landscape variables or species interactions, which likely supports the notion of multi-species convergence on productive prey sites, rather than competitive interactions.

Competition was more important in explaining spatial and temporal segregation among jaguars and pumas, than between either of these apex predators and ocelot. Otherwise, interspecific competition played an important role between ocelot and smaller sympatric cats [18], since both the local occupancy and circadian activity rhythms of ocelots affect jaguarundi and margay.

A recent global-scale study of co-occurrence of sympatric carnivores found that similar-sized species sharing the same temporal activity patterns and dietary habits were more likely to co-occur than expected by chance [7]. Although, the study used a categorization to describe general activity patterns and diet, not capturing variations on carnivores' behaviour at a particular study area. Indeed, our results showed that some spatial and temporal overlapping may occur, mainly between the three largest species, but go further assessing finer-scale of resource availability and diurnal rhythms, detecting niche partitioning in a local scale and differences in felids’ behaviour across study sites. In this paper, we highlight the importance of understanding the implications of interspecific interactions to conservation and management strategies, particularly in terms of rapidly declining carnivore populations, which may have major impacts on the diversity of lower trophic levels [2].

## Supporting information

S1 Table Prey species list and relative abundance index (images/100 ctdays) of small (< 15 Kg) and large prey (> 15Kg) of carnivores in our eight Neotropical study sites. (DOCX)

S2 Table Spearman’s rank correlation to test for collinearity among continuous covariates (ρ> 0.70). (DOCX)

S3 Table Model selection analysis for occupancy (Ψ) and detection probability (*p*) used to evaluate the effect of time (sampling period) and study site on the habitat use of three sympatric felids, the jaguar (*Panthera onca*), puma (*Puma concolor*) and ocelot (*Leopardus pardalis*) in Neotropical forests. (DOCX)

S4 Table Single-species detection models used to evaluate the effects of covariates on the detection probability (*p*) of three sympatric felids, the jaguar (*Panthera onca*), puma (*Puma concolor*) and ocelot (*Leopardus pardalis*) in Neotropical forests. Detection probability was modelled as a function of elevation, NDVI, study site (site), large prey availability (large) for jaguar and puma models and small prey availability (small) for ocelot models, or as a constant (p(.)).
(DOCX)

S5 Table Single-species occupancy models used to evaluate the effects of elevation (Elev.), distance to nearest water source (water), NDVI (ndvi), small prey’s availability (small) and large prey’s availability (large) on the habitat use of jaguar (*Panthera onca*), puma (*Puma concolor*) and ocelot (*Leopardus pardalis*) in Neotropical forests. (DOCX)

S6 Table Single-species occupancy models used to evaluate best habitat factors and species interactions. Occupancy probability was modelled as a function of elevation (Elev.), distance to water (water), NDVI (ndvi), small prey’s availability (small), large prey’s availability (large) and occupancy estimates of each cat species (jaguar, puma and ocelot).
(DOCX)

S7 Table Coefficient of overlap (Δ) with confidence intervals (CI lower/CI upper) and Watson’s two-sample test (two-sample *U*2) performed on pairwise comparisons between cat species per site. (DOCX)

S8 Table Coefficient of overlap (Δ1) with confidence intervals (CI lower/CI upper) and Watson’s two-sample test (two-sample *U*2) performed on pairwise comparisons between study sites. (DOCX)

S9 Table Differences in the daily activity level (*i. e.,* proportion of hours per day that an animal is active), standard errors (SE), Wald test (W) of Neotropical cats across the eight study sites (*Significant difference <0.05). (DOCX)
