# Supplementary material for: Prey availability and temporal partitioning modulate felid coexistence in Neotropical forests
Source: PLoS One. 2019 Mar 12;14(3):e0213671. doi: 10.1371/journal.pone.0213671 (PMC6413900; doi:10.1371/journal.pone.0213671)
Supplement: S1 Table — (DOCX) [file pone.0213671.s001.docx]

S1 Table – Prey species list and relative abundance index (images/100 ctdays) of small-bodied prey (< 15 Kg) and large-bodied prey (> 15Kg) of carnivores in our eight Neotropical forest study sites. Site codes: BCI - Barro Colorado Nature Monument, CAX - Caxiuanã National Forest, COU - Cocha Cashu - Manu National Park, CSN - Central Suriname Nature Reserve, MAN – Manaus, VB - Volcan Barva Transect, YAN - Yanachaga National Park, YAS - Yasuni Research Station.

| Species | BCI | CAX | COU | CSN | MAN | VB | YAN | YAS |
| --- | --- | --- | --- | --- | --- | --- | --- | --- |
| ***Small prey - mammals*** |  |  |  |  |  |  |  |  |
| *Cabassous centralis* | 0.13 | - | - | - | - | - | - | - |
| *Cabassous unicinctus* | - | 0.02 | - | - | 0.02 | - | 0.01 | - |
| *Caluromys derbianus* | - | - | - | - | - | 0.03 | - | - |
| *Cuniculus paca* | 8.27 | 2.79 | 7.55 | 3.45 | 2.63 | 3.36 | 12.83 | 8.59 |
| *Dasyprocta fuliginosa* | - | - | - | - | - | - | 6.46 | 15.13 |
| *Dasyprocta leporina* | - | 13.60 | - | 10.53 | 5.76 | - | - | - |
| *Dasyprocta punctata* | 52.67 | - | 9.93 | - | - | 2.93 | - | - |
| *Dasypus kappleri* | - | 2.74 | 0.37 | 1.30 | - | - | 0.36 | 1.36 |
| *Dasypus novemcinctus* | 3.96 | 2.21 | 1.11 | 2.27 | 3.09 | 1.45 | 2.44 | 4.10 |
| *Didelphis marsupialis* | 3.27 | 1.61 | 2.36 | 1.66 | 3.74 | 0.20 | 0.48 | 0.29 |
| *Marmosa demerarae* | - | 0.01 | - | - | 0.02 | - | - | - |
| *Marmosa murina* | - | - | - | 0.02 | - | - | - | - |
| *Marmosa regina* | - | - | 0.02 | - | - | - | - | - |
| *Marmosa robinsoni* | 0.03 | - | - | - | - | - | - | - |
| *Metachirus nudicaudatus* | 0.26 | 0.96 | - | 1.34 | 5.57 | - | 0.01 | 0.19 |
| *Monodelphis brevicaudata* | - | - | - | 0.01 | - | - | - | - |
| *Monodelphis glirina* | - | - | 0.13 | - | - | - | - | - |
| *Myoprocta acouchy* | - | - | - | 6.17 | 17.52 | - | - | - |
| *Myoprocta pratti* | - | - | 4.86 | - | - | - | - | 11.38 |
| *Nasua narica* | 4.92 | - | - | - | - | 0.30 | - | - |
| *Nasua nasua* | - | 0.73 | 0.23 | 0.08 | 0.15 | 0.00 | 1.29 | 0.58 |
| *Philander opossum* | 0.18 | - | 0.75 | 1.27 | 0.78 | 0.03 | - | - |
| *Proechimys brevicauda* | - | - | 12.48 | - | - | - | - | - |
| *Proechimys guyannensis* | - | - | - | 1.24 | - | - | - | - |
| *Proechimys semispinosus* | 3.87 | - | 0.02 | - | - | 0.01 | - | - |
| *Proechimys sp* | - | 0.30 | 2.63 | - | 0.07 | - | 0.22 | 0.11 |
| *Sciurus aestuans* | - | 0.03 | - | 0.07 | 0.09 | - | - | - |
| *Sciurus granatensis* | 0.93 | - | - | - | - | - | - | - |
| *Sciurus ignitus* | - | - | 2.08 | - | - | - | 0.01 | - |
| *Sciurus igniventris* | - | - | 0.01 | - | - | - | - | 2.19 |
| *Sciurus spadiceus* | - | - | 0.27 | - | - | - | 0.59 | - |
| *Sciurus variegatoides* | - | - | - | - | - | 0.01 | - | - |
| *Sylvilagus brasiliensis* | 0.13 | - | 0.49 | - | - | - | - | 0.18 |
| *Tamandua mexicana* | 0.92 | - | - | - | - | - | - | - |
| *Tamandua tetradactyla* | - | 0.19 | 0.08 | 0.04 | 0.13 | - | 0.07 | 0.19 |
| *Tylomys watsoni* | - | - | - | - | - | - | 0.24 | - |
| ***Small prey - birds*** |  |  |  |  |  |  |  |  |
| *Crax alector* | - | - | - | 2.30 | 1.39 | - | - | - |
| *Crax rubra* | 0.50 | - | - | - | - | 0.53 | - | - |
| *Crypturellus atrocapillus* | - | - | 0.03 | - | - | - | - | - |
| *Crypturellus bartletti* | - | - | 0.77 | - | - | - | - | 0.02 |
| *Crypturellus cinereus* | - | - | 0.19 | 0.04 | - | - | - | 0.02 |
| *Crypturellus soui* | - | - | 1.02 | 0.02 | - | - | 0.01 | 0.06 |
| *Crypturellus strigulosus* | - | 0.04 | - | - | - | - | - | - |
| *Crypturellus undulatus* | - | - | 0.17 | - | - | - | - | 0.01 |
| *Crypturellus variegatus* | - | 0.19 | 0.21 | 0.36 | 0.87 | - | - | 0.32 |
| *Geotrygon frenata* | - | - | - | - | - | - | 0.15 | - |
| *Geotrygon montana* | 0.43 | 0.05 | 0.15 | 0.37 | 1.65 | - | - | 1.34 |
| *Geotrygon saphirina* | - | - | - | - | - | - | - | 0.02 |
| *Geotrygon violacea* | - | - | 0.02 | 0.11 | - | - | - | - |
| *Leptotila cassini* | 0.93 | - | - | - | - | - | - | - |
| *Leptotila rufaxilla* | - | - | 1.11 | 0.51 | - | - | - | 0.13 |
| *Leptotila verreauxi* | 0.03 | - | - | - | 0.11 | - | - | 0.03 |
| *Mitu salvini* | - | - | - | - | - | - | - | 0.98 |
| *Mitu tuberosum* | - | 1.23 | 5.32 | - | - | - | 4.56 | - |
| *Odontophorus gujanensis* | 0.02 | - | - | 0.09 | 0.04 | - | - | 0.16 |
| *Odontophorus stellatus* | - | - | 0.50 | - | - | - | - | - |
| *Penelope jacquacu* | - | - | 0.25 | 0.05 | - | - | 0.19 | 0.37 |
| *Penelope marail* | - | - | - | 0.01 | - | - | - | - |
| *Penelope pileata* | - | 0.04 | - | - | - | - | - | - |
| *Penelope purpurascens* | 0.05 | - | - | - | - | - | - | - |
| *Penelope superciliaris* | - | 0.06 | - | - | - | - | - | - |
| *Pipile cumanensis* | - | - | 0.01 | - | - | - | - | - |
| *Pipile pipile* | - | - | - | - | - | - | - | 0.03 |
| *Psophia crepitans* | - | - | - | 5.19 | 4.59 | - | - | 7.32 |
| *Psophia leucoptera* | - | - | 7.97 | - | - | - | - | - |
| *Psophia viridis* | - | 3.48 | - | - | - | - | - | - |
| *Tinamus guttatus* | - | 0.10 | 0.06 | - | - | - | 0.05 | 0.08 |
| *Tinamus major* | 2.34 | 0.05 | 2.12 | 2.00 | 1.91 | - | 0.06 | 1.66 |
| *Tinamus tao* | - | 0.18 | 0.70 | - | - | - | 0.99 | - |
| *Small prey - Sub-total* | 83.86 | 30.59 | 65.96 | 40.47 | 50.13 | 8.85 | 31.03 | 56.82 |
| ***Large prey - mammals*** |  |  |  |  |  |  |  |  |
| *Hydrochoerus hydrochaeris* | - | - | 0.02 | - | - | - | - | - |
| *Hydrochoerus isthmius* | 0.01 | - | - | - | - | - | - | - |
| *Mazama americana* | - | 4.67 | 6.28 | 6.69 | 1.96 | - | 1.01 | 9.57 |
| *Mazama nemorivaga* | - | 2.85 | - | 1.42 | 1.33 | - | - | 2.80 |
| *Mazama temama* | 3.32 | - | - | - | - | 2.34 | - | - |
| *Myrmecophaga tridactyla* | - | 0.57 | 0.51 | 0.42 | 0.28 | - | 0.15 | 0.59 |
| *Odocoileus virginianus* | 1.64 | - | - | 0.04 | - | 0.10 | - | - |
| *Pecari tajacu* | 9.31 | 1.82 | 2.77 | 1.49 | 1.96 | 14.53 | 0.64 | 8.80 |
| *Priodontes maximus* | - | 0.11 | 0.36 | 0.42 | - | - | - | 0.30 |
| *Tapirus bairdii* | 0.01 | - | - | - | - | 2.01 | - | - |
| *Tapirus terrestris* | - | 0.90 | 2.87 | 2.58 | 0.41 | - | 1.90 | 2.61 |
| *Tayassu pecari* | - | 0.18 | 0.57 | 0.23 | 0.15 | - | - | 2.28 |
| *LARGE PREY SUB-TOTAL* | 14.30 | 11.09 | 13.37 | 13.29 | 6.09 | 18.98 | 3.70 | 26.94 |
| **TOTAL** | **98.16** | **41.68** | **79.34** | **53.76** | **56.22** | **27.83** | **34.73** | **83.76** |
