# Supplementary material for: Prey availability and temporal partitioning modulate felid coexistence in Neotropical forests
Source: PLoS One. 2019 Mar 12;14(3):e0213671. doi: 10.1371/journal.pone.0213671 (PMC6413900; doi:10.1371/journal.pone.0213671)
Supplement: S2 Table — (DOCX) [file pone.0213671.s002.docx]

S2 Table – Spearman’s rank correlation to test for collinearity among continuous covariates (ρ > 0.70).

|  | Small prey | Large prey | Elevation range | Dist.to water | NDVI | Slope |
| --- | --- | --- | --- | --- | --- | --- |
| Small prey | 1 | 0.55 | -0.11 | -0.01 | 0.00 | -0.13 |
| Large prey | 0.55 | 1 | -0.16 | 0.00 | -0.03 | -0.16 |
| Elevation range | -0.11 | -0.16 | 1 | -0.07 | -0.10 | 0.86 |
| Distance to water | -0.01 | 0.00 | -0.07 | 1 | -0.07 | -0.11 |
| NDVI | 0.00 | -0.03 | -0.10 | -0.07 | 1 | -0.11 |
| Slope | -0.13 | -0.16 | **0.86** | -0.11 | -0.11 | 1 |
