# Supplementary material for: Prey availability and temporal partitioning modulate felid coexistence in Neotropical forests
Source: PLoS One. 2019 Mar 12;14(3):e0213671. doi: 10.1371/journal.pone.0213671 (PMC6413900; doi:10.1371/journal.pone.0213671)
Supplement: S3 Table — (DOCX) [file pone.0213671.s003.docx]

S3 Table - Model selection analysis for occupancy (Ψ) and detection probability (*p*) used to evaluate the effect of time (sampling period) and study site on the habitat use of three sympatric felids, the jaguar (*Panthera onca*), the puma (*Puma concolor*) and the ocelot (*Leopardus pardalis*) in Neotropical forests.

| Models |  |  |  |  |  |  |
| --- | --- | --- | --- | --- | --- | --- |
| **Jaguar** | **K** | **AIC** | **∆AIC** | **AIC_wt_** | **CumltvWt** | **Rsq** |
| Ψ(.)p(.) | 2 | 1824.31 | 0 | 0.50 | 0.5 | 0.00 |
| Ψ(site)p(.) | 7 | 1824.58 | 0.27 | 0.43 | 0.93 | 0.01 |
| Ψ(time)p(.) | 28 | 1828.79 | 4.48 | 0.05 | 0.98 | 0.04 |
| Ψ(.)p(time) | 28 | 1831.61 | 7.3 | 0.01 | 0.99 | 0.04 |
| Ψ(site)p(site) | 12 | 1833.3 | 8.99 | 0.01 | 1 | 0.01 |
| Ψ(time)p(site) | 33 | 1837.32 | 13.01 | 0.00 | 1 | 0.05 |
| Ψ(time)p(time) | 54 | 1860.46 | 36.15 | 0.00 | 1 | 0.06 |
| **Puma** | **K** | **QAIC** | **∆QAIC** | **QAIC_Wt_** | **Cum.Wt** | **Quasi.LL** |
| Ψ(site)p(.) | 8 | 1102.06 | 0.00 | 0.87 | 0.87 | -543.03 |
| Ψ(site)p(site) | 13 | 1106.83 | 4.78 | 0.08 | 0.95 | -540.42 |
| Ψ(.)p(.) | 3 | 1107.62 | 5.56 | 0.05 | 1.00 | -550.81 |
| Ψ(time)p(.) | 29 | 1133.97 | 31.91 | 0.00 | 1.00 | -537.98 |
| Ψ(.)p(time) | 29 | 1135.39 | 33.33 | 0.00 | 1.00 | -538.69 |
| Ψ(time)p(site) | 34 | 1138.75 | 36.69 | 0.00 | 1.00 | -535.37 |
| **Ocelot** | **K** | **QAIC** | **∆QAIC** | **QAIC_Wt_** | **Cum.Wt** | **Quasi.LL** |
| Ψ(site)p(site) | 17 | 4816.73 | 0.00 | 1.00 | 1.00 | -2391.36 |
| Ψ(.)p(time) | 39 | 4833.67 | 16.95 | 0.00 | 1.00 | -2377.84 |
| Ψ(time)p(site) | 46 | 4842.27 | 25.54 | 0.00 | 1.00 | -2375.13 |
| Ψ(site)p(.) | 10 | 4869.07 | 52.34 | 0.00 | 1.00 | -2424.53 |
| Ψ(time)p(time) | 75 | 4886.52 | 69.79 | 0.00 | 1.00 | -2368.26 |
| Ψ(time)p(.) | 39 | 4895.88 | 79.16 | 0.00 | 1.00 | -2408.94 |
| Ψ(.)p(.) | 3 | 5016.47 | 199.75 | 0.00 | 1.00 | -2505.24 |
