# Supplementary material for: Prey availability and temporal partitioning modulate felid coexistence in Neotropical forests
Source: PLoS One. 2019 Mar 12;14(3):e0213671. doi: 10.1371/journal.pone.0213671 (PMC6413900; doi:10.1371/journal.pone.0213671)
Supplement: S4 Table — Detection probability was modelled as a function of elevation, NDVI, study site (site), large prey availability (large) for jaguar and puma models and small prey availability (small) for ocelot models, or as a constant (p(.)). (DOCX) [file pone.0213671.s004.docx]

S4 Table - Single-species detection models used to evaluate the effects of covariates on the detection probability (*p*) of three sympatric felids, the jaguar (*Panthera onca*), puma (*Puma concolor*) and ocelot (*Leopardus pardalis*) in Neotropical forests. Detection probability was modelled as a function of elevation range (elevation), NDVI (ndvi), study site (site), large-bodied prey availability (large) for jaguar and puma models and small-bodied prey availability (small) for ocelot models, or as a constant (p(.)).

| **Models** | | | | | **Beta estimates (±SE)** | | |
| --- | --- | --- | --- | --- | --- | --- | --- |
| **JAGUAR** | **K** | **AIC** | **∆AIC** | **AICwt** | **Large prey** | **Elev.** | **NDVI** |
| ψ(.)p(site+large) | 8 | 1816.89 | 0 | 0.42 | 0.44 (0.14) | - | - |
| ψ(.)p(large+elevation) | 4 | 1818.45 | 1.57 | 0.19 | 0.30 (0.12) | -0.19 (0.12) | - |
| ψ(.)p(large) | 3 | 1819.28 | 2.4 | 0.13 | 0.33 (0.11) | - | - |
| ψ(.)p(large+elevation+site+ndvi) | 10 | 1819.91 | 3.03 | 0.09 | 0.45 (0.14) | 0.16 (0.18) | -0.04 (0.09) |
| ψ(.)p(large+ndvi) | 4 | 1820.73 | 3.84 | 0.06 | 0.34 (0.11) | - | 0.06 (0.08) |
| ψ(.)p(elevation) | 3 | 1821.36 | 4.47 | 0.05 | - | -0.24 (0.12) | - |
| ψ(.)p(elevation+ndvi) | 4 | 1823.2 | 6.31 | 0.02 | - | -0.24 (0.12) | 0.03 (0.08) |
| ψ(.)p(site) | 7 | 1823.99 | 7.1 | 0.01 | - | - | - |
| ψ(.)p(.) | 2 | 1824.31 | 7.42 | 0.01 | - | - | - |
| ψ(.)p(site+elevation) | 8 | 1825.13 | 8.24 | 0.01 | - | -0.15 (0.17) | - |
| ψ(.)p(site+ndvi) | 8 | 1825.94 | 9.06 | 0.00 | - | - | -0.02 (0.09) |
| ψ(.)p(ndvi) | 3 | 1825.98 | 9.1 | 0.00 | - | - | 0.05 (0.08) |
| **PUMA** | **K** | **QAIC** | **∆QAIC** | **QAICWt** | **Large prey** | **Elev.** | **NDVI** |
| ψ(.)p(large+elevation) | 5 | 982.39 | 0.00 | 0.31 | 0.33 (0.11) | -0.36 (0.14) | - |
| ψ(.)p(elevation) | 4 | 984.05 | 1.66 | 0.14 | - | -0.41 (0.14) | - |
| ψ(.)p(large) | 4 | 984.56 | 2.17 | 0.11 | 0.39 (0.11) | - | - |
| ψ(.)p(site+large) | 9 | 984.67 | 2.28 | 0.10 | 0.36 (0.13) | - | - |
| ψ(.)p(elevation+ndvi) | 5 | 985.07 | 2.68 | 0.08 | - | -0.41 (0.14) | 0.12 (0.09) |
| ψ(.)p(large+ndvi) | 5 | 985.17 | 2.78 | 0.08 | 0.39 (0.10) | - | 0.14 (0.09) |
| ψ(.)p(site) | 8 | 985.96 | 3.57 | 0.05 | - | - | - |
| ψ(.)p(site+ndvi) | 9 | 986.79 | 4.40 | 0.03 | - | - | 0.14 (0.10) |
| ψ(.)p(site+elevation) | 9 | 986.96 | 4.57 | 0.03 | - | -0.27 (0.20) | - |
| ψ(.)p(large+elevation+site+ndvi) | 11 | 987.05 | 4.66 | 0.03 | 0.34 (0.12) | -0.24 (0.20) | 0.11 (0.10) |
| ψ(.)p(.) | 3 | 988.05 | 5.66 | 0.02 | - | - | - |
| ψ(.)p(ndvi) | 4 | 988.76 | 6.37 | 0.01 | - | - | 0.14 (0.09) |
| **OCELOT** | **K** | **AIC** | **∆AIC** | **AICwt** | **Small prey** | **Elev.** | **NDVI** |
| ψ(.)p(site+small) | 8 | 4663.65 | 0 | 0.67 | 0.14 (0.04) | - | - |
| ψ(.)p(small+elevation+site+ndvi) | 10 | 4665.12 | 1.47 | 0.32 | 0.14 (0.04) | 0.13 (0.10) | -0.03 (0.05) |
| ψ(.)p(site+elevation) | 8 | 4673.25 | 9.6 | 0.01 | - | 0.14 (0.10) | - |
| ψ(.)p(site) | 7 | 4673.38 | 9.73 | 0.01 | - | - | - |
| ψ(.)p(site+ndvi) | 8 | 4674.83 | 11.17 | 0.00 | - | - | -0.04 (0.05) |
| ψ(.)p(small+elevation) | 4 | 4817.44 | 153.79 | 0.00 | 0.28 (0.05) | -0.11 (0.06) | - |
| ψ(.)p(small) | 3 | 4818.82 | 155.16 | 0.00 | 0.30 (0.05) | - | - |
| ψ(.)p(small+ndvi) | 4 | 4820.78 | 157.12 | 0.00 | 0.30 (0.05) | - | -0.01 (0.05) |
| ψ(.)p(elevation) | 3 | 4857.68 | 194.03 | 0.00 | - | -0.15 (0.06) | - |
| ψ(.)p(elevation+ndvi) | 4 | 4859.62 | 195.97 | 0.00 | - | -0.15 (0.06) | -0.01 (0.05) |
| ψ(.)p(.) | 2 | 4862.87 | 199.22 | 0.00 | - | - | - |
| ψ(.)p(ndvi) | 3 | 4864.87 | 201.22 | 0.00 | - | - | 0.00 (0.05) |
