# Supplementary material for: Prey availability and temporal partitioning modulate felid coexistence in Neotropical forests
Source: PLoS One. 2019 Mar 12;14(3):e0213671. doi: 10.1371/journal.pone.0213671 (PMC6413900; doi:10.1371/journal.pone.0213671)
Supplement: S5 Table — (DOCX) [file pone.0213671.s005.docx]

S5 Table - Single-species occupancy models used to evaluate the effects of elevation (Elev.), distance to nearest water source (water), NDVI (ndvi), small-bodied prey’s availability (small) and large-bodied prey’s availability (large) on the habitat use of jaguar (*Panthera onca*), puma (*Puma concolor*) and ocelot (*Leopardus pardalis*) in Neotropical forests.

| **Models** |  |  |  |  | **Beta estimates (±SE)** | | | | |
| --- | --- | --- | --- | --- | --- | --- | --- | --- | --- |
| **Jaguar** | **K** | **AIC** | **∆AIC** | **AIC_Wt_** | **Elev.** | **Dist. Water** | **NDVI** | **Large prey** | **Small prey** |
| ψ(large+water)p(large+site) | 10 | 1812.13 | 0 | 0.34 | - | -0.28 (0.17) | - | 1.54 (0.54) | - |
| ψ(large)p(large+site) | 9 | 1813.02 | 0.89 | 0.21 | - | - | - | 1.42 (0.53) | - |
| ψ(large+small)p(large+site) | 10 | 1814.41 | 2.29 | 0.11 | - | - | - | 1.38 (0.53) | 0.14 (0.18) |
| ψ(large+elevation)p(large+site) | 10 | 1814.63 | 2.5 | 0.10 | -0.14 (0.21) | - | - | 1.42 (0.54) | - |
| ψ(large+ndvi)p(large+site) | 10 | 1814.7 | 2.57 | 0.09 | - | - | 0.09 (0.15) | 1.49 (0.55) | - |
| ψ(.)p(large+site) | 8 | 1816.89 | 4.76 | 0.03 | - | - | - | - | - |
| ψ(global)p(large+site) | 13 | 1817.01 | 4.88 | 0.03 | -0.09 (0.22) | -0.27 (0.17) | 0.09 (0.15) | 1.55 (0.55) | 0.13 (0.19) |
| ψ(water)p(large+site) | 9 | 1817.52 | 5.39 | 0.02 | - | -0.19 (0.16) | - | - | - |
| ψ(elevation)p(large+site) | 9 | 1818.4 | 6.28 | 0.01 | -0.18 (0.24) | - | - | - | - |
| ψ(small)p(large+site) | 9 | 1818.74 | 6.61 | 0.01 | - | - | - | - | -0.03 (0.08) |
| ψ(ndvi)p(large+site) | 9 | 1818.82 | 6.69 | 0.01 | - | - | -0.04 (0.15) | - | - |
| ψ(water+elevation)p(large+site) | 10 | 1819.15 | 7.02 | 0.01 | -0.17 (0.25) | -0.18 (0.16) | - | - | - |
| ψ(water+ndvi)p(large+site) | 10 | 1819.45 | 7.32 | 0.01 | - | -0.19 (0.16) | -0.04 (0.14) | - | - |
| ψ(elevation+small)p(large+site) | 10 | 1820.22 | 8.1 | 0.01 | -0.19 (0.24) | - | - | - | -0.03 (0.08) |
| ψ(elevation+ndvi)p(large+site) | 10 | 1820.31 | 8.18 | 0.01 | -0.19 (0.24) | - | -0.04 (0.15) | - | - |
| ψ(water+small)p(large+site) | 10 | 1824.03 | 11.9 | 0.00 | - | -0.20 (0.17) | - | - | 0.27 (0.24) |
| ψ(small+ndvi)p(large+site) | 10 | 1825.46 | 13.33 | 0.00 | - | - | -0.02 (0.15) | - | 0.25 (0.23) |
| **Puma** | **K** | **QAIC** | **∆QAIC** | **QAIC_Wt_** | **Elev.** | **Dist. Water** | **NDVI** | **Large prey** | **Small prey** |
| ψ(water)p(large+elevation) | 10 | 521.67 | 0.00 | 0.19 | - | -0.21 (0.15) | - | - | - |
| ψ(.)p(large+elevation) | 11 | 522.58 | 0.92 | 0.12 | - | - | - | - | - |
| ψ(water+ndvi)p(large+elevation) | 11 | 523.26 | 1.59 | 0.09 | - | -0.21 (0.15) | 0.15 (0.13) | - | - |
| ψ(ndvi)p(large+elevation) | 11 | 523.38 | 1.71 | 0.08 | - | - | 0.16 (0.13) | - | - |
| ψ(water+elevation)p(large+elevation) | 11 | 523.61 | 1.94 | 0.07 | -0.24 (0.23) | -0.20 (0.15) | - | - | - |
| ψ(elevation)p(large+elevation) | 11 | 523.62 | 1.95 | 0.07 | -0.27 (0.22) | - | - | - | - |
| ψ(small)p(large+elevation) | 12 | 524.28 | 2.61 | 0.05 | - | - | - | - | -0.02 (0.08) |
| ψ(large+water)p(large+elevation) | 12 | 524.38 | 2.72 | 0.05 | - | -0.18 (0.16) | - | 0.38 (0.32) | - |
| ψ(large)p(large+elevation) | 12 | 524.50 | 2.83 | 0.05 | - | - | - | 0.45 (0.36) | - |
| ψ(elevation+ndvi)p(large+elevation) | 12 | 524.57 | 2.90 | 0.04 | -0.25 (0.23) | - | 0.15 (0.13) | - | - |
| ψ(water+small)p(large+elevation) | 12 | 524.97 | 3.30 | 0.04 | - | -0.21 (0.16) | - | - | 0.24 (0.22) |
| ψ(large+ndvi)p(large+elevation) | 12 | 525.21 | 3.54 | 0.03 | - | - | 0.17 (0.14) | 0.44 (0.37) | - |
| ψ(elevation+small)p(large+elevation) | 12 | 525.22 | 3.56 | 0.03 | -0.27 (0.22) | - | - | - | -0.02 (0.08) |
| ψ(large+small)p(large+elevation) | 12 | 525.29 | 3.62 | 0.03 | - | - | - | 0.41 (0.36) | 0.20 (0.19) |
| ψ(large+elevation)p(large+elevation) | 12 | 525.35 | 3.68 | 0.03 | -0.27 (0.21) | - | - | 0.45 (0.37) | - |
| ψ(small+ndvi)p(large+elevation) | 12 | 525.49 | 3.82 | 0.03 | - | - | 0.17 (0.13) | - | 0.23 (0.22) |
| ψ(global)p(large+elevation) | 15 | 529.95 | 8.28 | 0.00 | -0.24 (0.22) | -0.17 | 0.16 (0.13) | 0.35 (0.33) | 0.20 (0.20) |
| **Ocelot** | **K** | **QAIC** | **∆QAIC** | **QAIC_Wt_** | **Elev.** | **Dist. Water** | **NDVI** | **Large prey** | **Small prey** |
| ψ(small)p(site+small) | 10 | 3549.98 | 0.00 | 0.29 | - | - | - | - | 0.77 (0.27) |
| ψ(small+elevation)p(site+small) | 11 | 3550.10 | 0.11 | 0.28 | 0.32 (0.26) | - | - | - | 0.85 (0.29) |
| ψ(small+large)p(site+small) | 11 | 3551.34 | 1.36 | 0.15 | - | - | - | -0.20 (0.21) | 0.84 (0.29) |
| ψ(small+ndvi)p(site+small) | 11 | 3551.46 | 1.48 | 0.14 | - | - | -0.10 (0.13) | - | 0.79 (0.27) |
| ψ(small+water)p(site+small) | 11 | 3551.98 | 2.00 | 0.11 | - | -0.01 (0.13) | - | - | 0.77 (0.27) |
| ψ(global)p(site+small) | 14 | 3555.21 | 5.22 | 0.02 | 0.31 (0.28) | -0.03 (0.14) | -0.07 (0.12) | -0.20 (0.21) | 0.92 (0.31) |
| ψ(.)p(site+small) | 9 | 3560.69 | 10.71 | 0.00 | - | - | - | - | - |
| ψ(elevation)p(site+small) | 10 | 3562.13 | 12.15 | 0.00 | 0.20 (0.28) | - | - | - | - |
| ψ(large)p(site+small) | 10 | 3562.24 | 12.26 | 0.00 | - | - | - | 0.07 (0.13) | - |
| ψ(ndvi)p(site+small) | 10 | 3562.40 | 12.42 | 0.00 | - | - | -0.08 (0.14) | - | - |
| ψ(water)p(site+small) | 10 | 3562.69 | 12.70 | 0.00 | - | 0.01 (0.14) | - | - | - |
| ψ(elevation+large)p(site+small) | 11 | 3563.65 | 13.66 | 0.00 | 0.20 (0.27) | - | - | 0.08 (0.14) | - |
| ψ(elevation+ndvi)p(site+small) | 11 | 3563.93 | 13.94 | 0.00 | 0.19 (0.28) | - | -0.07 (0.14) | - | - |
| ψ(large+ndvi)p(site+small) | 11 | 3563.93 | 13.94 | 0.00 | - | - | -0.08 (0.14) | 0.08 (0.14) | - |
| ψ(water+elevation)p(site+small) | 11 | 3564.13 | 14.14 | 0.00 | 0.20 (0.28) | -0.01 (0.14) | - | - | - |
| ψ(water+large)p(site+small) | 11 | 3564.23 | 14.25 | 0.00 | - | 0.02 (0.14) | - | 0.08 (0.14) | - |
| ψ(water+ndvi)p(site+small) | 11 | 3564.36 | 14.38 | 0.00 | - | 0.03 (0.15) | -0.09 (0.16) | - | - |
