# Supplementary material for: Prey availability and temporal partitioning modulate felid coexistence in Neotropical forests
Source: PLoS One. 2019 Mar 12;14(3):e0213671. doi: 10.1371/journal.pone.0213671 (PMC6413900; doi:10.1371/journal.pone.0213671)
Supplement: S6 Table — Occupancy probability was modelled as a function of elevation (Elev.), distance to water (water), NDVI (ndvi), small prey’s availability (small), large prey’s availability (large) and occupancy estimates of each cat species (jaguar, puma and ocelot). (DOCX) [file pone.0213671.s006.docx]

| S6 Table - Single-species occupancy models used to evaluate best habitat factors and species interactions. Occupancy probability (Ψ ) was modelled as a function of elevation (Elev.), distance to the nearest water source (water), NDVI (ndvi), small-bodied prey’s availability (small), large-bodied prey’s availability (large) and occupancy estimates of each cat species (jaguar, puma and ocelot). | | | | | | | | | | | |
| --- | --- | --- | --- | --- | --- | --- | --- | --- | --- | --- | --- |
| **Models** |  |  |  |  | **Beta estimates (±SE)** | | | | | | |
| **Jaguar** | **K** | **AIC** | **∆AIC** | **AIC_wt_** | **Elev.** | **Dist. Water** | **NDVI** | **Large prey** | **Small prey** | **Puma** | **Ocelot** |
| ψ(puma+large)p(large+site) | 10 | 1811.85 | 0 | 0.33 | - | - | - | 1.60 (0.55) | - | 0.30 (0.18) | - |
| ψ(large+water)p(large+site) | 10 | 1812.13 | 0.28 | 0.29 | - | -0.28 (0.17) | - | 1.54 (0.54) | - | - | - |
| ψ(large)p(large+site) | 9 | 1813.02 | 1.17 | 0.19 | - | - | - | 1.42 (0.54) | - | - | - |
| ψ(ocelot+large)p(large+site) | 10 | 1814.88 | 3.03 | 0.07 | - | - | - | 1.40 (0.53) | - | - | 0.06 (0.15) |
| ψ(.)p(large+site) | 8 | 1816.89 | 5.04 | 0.03 | - | - | - | - | - | - | - |
| ψ(puma)p(large+site) | 9 | 1817.86 | 6.01 | 0.02 | - | - | - | - | - | 0.17 (0.16) | - |
| ψ(ocelot)p(large+site) | 9 | 1818.46 | 6.61 | 0.01 | - | - | - | - | - | - | 0.09 (0.14) |
| ψ(global)p(large+site) | 15 | 1819 | 7.15 | 0.01 | 0.38 (1.02) | 2.63 (4.37) | -0.91 (1.25) | 1.43 (0.64) | 8.86 (7.76) | 3.18 (4.62) | -5.87 (5.03) |
| ψ(ocelot+water)p(large+site) | 10 | 1819.04 | 7.19 | 0.01 | - | -0.19 (0.16) | - | - | - | - | 0.10 (0.14) |
| ψ(puma+ocelot)p(large+site) | 10 | 1819.37 | 7.52 | 0.01 | - | - | - | - | - | 0.17 (0.16) | 0.10 (0.14) |
| ψ(puma+elevation)p(large+site) | 10 | 1819.38 | 7.53 | 0.01 | -0.19 (0.24) | - | - | - | - | 0.17 (0.16) | - |
| ψ(puma+water)+p(large+site) | 10 | 1819.42 | 7.57 | 0.01 | - | -0.35 (0.55) | - | - | - | -0.17 (0.56) | - |
| ψ(puma+ndvi)+p(large+site) | 10 | 1819.5 | 7.65 | 0.01 | - | - | -0.09 (0.15) | - | - | 0.20 (0.17) | - |
| ψ(ocelot+elevation)p(large+site) | 10 | 1819.98 | 8.13 | 0.01 | -0.18 (0.24) | - | - | - | - | - | 0.09 (0.14) |
| ψ(ocelot+ndvi)p(large+site) | 10 | 1820.4 | 8.55 | 0.00 | - | - | -0.03 (0.15) | - | - | - | 0.09 (0.14) |
| ψ(ocelot+small)p(large+site) | 10 | 1822.82 | 10.97 | 0.00 | - | - | - | - | 2.56 (1.85) | - | -1.67 (1.23) |
| ψ(puma+small)p(large+site) | 10 | 1824.29 | 12.44 | 0.00 | - | - | - | - | 0.27 (0.24) | 0.19 (0.17) | - |
| **Puma** | **K** | **QAIC** | **∆QAIC** | **QAIC_wt_** | **Elev.** | **Dist. Water** | **NDVI** | **Large prey** | **Small prey** | **Jaguar** | **Ocelot** |
| ψ(.)p(large+elevation) | 5 | 664.24 | 0.00 | 0.19 | - | - | - | - | - | - | - |
| ψ(water)p(large+elevation) | 6 | 664.69 | 0.46 | 0.15 | - | -0.29 (0.14) | - | - | - | - | - |
| ψ(ndvi)p(large+elevation) | 6 | 665.65 | 1.41 | 0.09 | - | - | 0.16 (0.12) | - | - | - | - |
| ψ(jaguar)p(large+elevation) | 6 | 665.92 | 1.69 | 0.08 | - | - | - | - | - | 0.32 (0.18) | - |
| ψ(ocelot)p(large+elevation) | 6 | 665.99 | 1.75 | 0.08 | - | - | - | - | - | - | 0.10 (0.12) |
| ψ(water+ndvi)p(large+elevation) | 7 | 666.19 | 1.96 | 0.07 | - | -0.28 (0.14) | 0.15 (0.12) | - | - | - | - |
| ψ(water+elevation)p(large+elevation) | 7 | 666.62 | 2.39 | 0.06 | 0.25 (0.54) | -0.29 (0.14) | - | - | - | - | - |
| ψ(jaguar+water)+p(large+elevation) | 7 | 667.10 | 2.86 | 0.05 | - | -0.22 (0.14) | - | - | - | 0.21 (0.22) | - |
| ψ(jaguar+ndvi)+p(large+elevation) | 7 | 667.29 | 3.06 | 0.04 | - | - | 0.18 (0.13) | - | - | 0.34 (0.18) | - |
| ψ(jaguar+large)p(large+elevation) | 7 | 667.43 | 3.19 | 0.04 | - | - | - | -0.32 (0.39) | - | 0.32 (0.28) | - |
| ψ(jaguar+small)p(large+elevation) | 7 | 667.65 | 3.41 | 0.03 | - | - | - | - | 0.16 (0.17) | 0.32 (0.17) | - |
| ψ(jaguar+ocelot)p(large+elevation) | 7 | 667.65 | 3.42 | 0.03 | - | - | - | - | - | 0.30 (0.18) | 0.11 (0.13) |
| ψ(jaguar+elevation)p(large+elevation) | 7 | 667.91 | 3.67 | 0.03 | 0.11 (0.51) | - | - | - | - | 0.32 (0.18) | - |
| ψ(ocelot+elevation)p(large+elevation) | 7 | 667.95 | 3.72 | 0.03 | 0.16 (0.52) | - | - | - | - | - | 0.10 (0.12) |
| ψ(ocelot+large)p(large+elevation) | 7 | 668.28 | 4.04 | 0.03 | - | - | - | 0.43 (0.32) | - | - | 0.13 (0.13) |
| ψ(ocelot+ndvi)p(large+elevation) | 7 | 673.63 | 9.39 | 0.00 | - | - | -26.22 (22.56) | - | - | - | 4.76 (3.36) |
| ψ(ocelot+water)p(large+elevation) | 7 | 673.68 | 9.44 | 0.00 | - | 68.27 (80.56) | - | - | - | - | 3.49 (4.87) |
| ψ(ocelot+small)p(large+elevation) | 7 | 675.26 | 11.02 | 0.00 | - | - | - | - | 9.26 (39.79) | - | -10.16 (41.61) |
| ψ(global)p(large+elevation) | 12 | 675.34 | 11.10 | 0.00 | 0.09 (0.59) | -0.30 (0.17) | 0.16 (0.13) | -0.10 (0.34) | -0.11 (0.55) | 0.03 (0.30) | 0.22 (0.45) |
| **Ocelot** | **K** | **QAIC** | **∆QAIC** | **QAIC_wt_** | **Elev.** | **Dist. Water** | **NDVI** | **Large prey** | **Small prey** | **Jaguar** | **Puma** |
| ψ(small)p(site+small) | 10 | 3196.69 | 0.00 | 0.25 | - | - | - | - | 0.77 (0.27) | - | - |
| ψ(small+elevation)p(site+small) | 11 | 3197.00 | 0.30 | 0.22 | 0.32 (0.26) | - | - | - | 0.85 (0.29) | - | - |
| ψ(small+large)p(site+small) | 11 | 3198.11 | 1.42 | 0.12 | - | - | - | -0.20 (0.21) | 0.84 (0.29) | - | - |
| ψ(small+ndvi)p(site+small) | 11 | 3198.22 | 1.53 | 0.12 | - | - | -0.10 (0.13) | - | 0.79 (0.27) | - | - |
| ψ(jaguar+small)p(site+small) | 11 | 3198.68 | 1.99 | 0.09 | - | - | - | - | 0.78 (0.28) | -0.01 (0.12) | - |
| ψ(puma+small)p(site+small) | 11 | 3198.69 | 2.00 | 0.09 | - | - | - | - | 0.77 (0.27) | - | -0.01 (0.14) |
| ψ(small+water)p(site+small) | 11 | 3198.69 | 2.00 | 0.09 | - | -0.01 (0.13) | - | - | 0.77 (0.27) | - | - |
| ψ(global)p(site+small) | 16 | 3203.55 | 6.86 | 0.01 | 0.68 (0.46) | -2.09 (2.28) | 0.54 (0.64) | -0.71 (0.28) | 1.01 (0.33) | 0.36 (0.18) | -2.29 (2.44) |
| ψ(.)p(site+small) | 9 | 3206.13 | 9.43 | 0.00 | - | - | - | - | - | - | - |
| ψ(jaguar)p(site+small) | 10 | 3207.78 | 11.09 | 0.00 | - | - | - | - | - | 0.09 (0.13) | - |
| ψ(puma)p(site+small) | 10 | 3208.10 | 11.41 | 0.00 | - | - | - | - | - | - | -0.03 (0.15) |
| ψ(jaguar+elevation)p(site+small) | 11 | 3209.20 | 12.50 | 0.00 | 0.21 (0.27) | - | - | - | - | 0.10 (0.13) | - |
| ψ(jaguar+ndvi)p(site+small) | 11 | 3209.52 | 12.83 | 0.00 | - | - | -0.08 (0.14) | - | - | 0.09 (0.13) | - |
| ψ(puma+elevation)p(site+small) | 11 | 3209.61 | 12.91 | 0.00 | 0.19 (0.27) | - | - | - | - | - | -0.03 (0.16) |
| ψ(jaguar+large)p(site+small) | 11 | 3209.62 | 12.93 | 0.00 | - | - | - | 0.05 (0.12) | - | 0.06 (0.15) | - |
| ψ(jaguar+puma)p(site+small) | 11 | 3209.64 | 12.95 | 0.00 | - | - | - | - | - | 0.11 (0.14) | -0.07 (0.17) |
| ψ(puma+large)p(site+small) | 11 | 3209.68 | 12.98 | 0.00 | - | - | - | 0.08 (0.14) | - | - | -0.04 (0.16) |
| ψ(jaguar+water)p(site+small) | 11 | 3209.72 | 13.02 | 0.00 | - | 0.05 (0.15) | - | - | - | 0.11 (0.14) | - |
| ψ(puma+ndvi)p(site+small) | 11 | 3209.85 | 13.15 | 0.00 | - | - | -0.08 (0.15) | - | - | - | -0.03 (0.16) |
| ψ(puma+water)p(site+small) | 11 | 3209.98 | 13.29 | 0.00 | - | -0.17 (0.43) | - | - | - | - | -0.21 (0.47) |
