# Supplementary material for: Prey availability and temporal partitioning modulate felid coexistence in Neotropical forests
Source: PLoS One. 2019 Mar 12;14(3):e0213671. doi: 10.1371/journal.pone.0213671 (PMC6413900; doi:10.1371/journal.pone.0213671)
Supplement: S7 Table — (DOCX) [file pone.0213671.s007.docx]

| S7 Table - Coefficient of overlap (Δ) with confidence intervals (CI lower/CI upper) and Watson’s two-sample test (two-sample *U*2) performed on pairwise comparisons between cat species per site. | | | | | | |
| --- | --- | --- | --- | --- | --- | --- |
| Site | Species | Coefficient of Overlap | | | Watson's Two-Sample Test | |
|  |  | Δ | CI lower | CI upper | U² | P value |
| BCI | Ocelot *vs* Jaguarundi | 0.436 | 0.274 | 0.598 | 0.3895 | < 0.001 |
| CAX | Jaguar *vs* Puma | 0.763 | 0.586 | 0.875 | 0.1964 | < 0.05 |
|  | Jaguar *vs* Ocelot | 0.645 | 0.559 | 0.918 | 0.2255 | < 0.05 |
|  | Puma *vs* Ocelot | 0.731 | 0.593 | 0.885 | 0.2053 | < 0.05 |
|  | Ocelot *vs* Margay | 0.741 | 0.578 | 0.876 | 0.0641 | ns |
| CSN | Jaguar *vs* Puma | 0.798 | 0.668 | 0.906 | 0.0814 | ns |
|  | Jaguar *vs* Ocelot | 0.823 | 0.717 | 0.915 | 0.1564 | ns |
|  | Puma *vs* Ocelot | 0.634 | 0.520 | 0.747 | 0.4759 | < 0.001 |
|  | Ocelot *vs* Jaguarundi | 0.484 | 0.378 | 0.588 | 0.8492 | < 0.001 |
|  | Ocelot *vs* Margay | 0.669 | 0.556 | 0.773 | 0.5277 | < 0.001 |
|  | Jaguarundi *vs* Margay | 0.197 | 0.091 | 0.304 | 1.5352 | < 0.001 |
| COU | Jaguar *vs* Puma | 0.670 | 0.535 | 0.810 | 0.3866 | < 0.001 |
|  | Jaguar *vs* Ocelot | 0.687 | 0.579 | 0.789 | 0.5393 | < 0.001 |
|  | Puma *vs* Ocelot | 0.662 | 0.557 | 0.770 | 0.7369 | < 0.001 |
|  | Ocelot *vs* Jaguarundi | 0.360 | 0.214 | 0.518 | 0.6856 | < 0.001 |
|  | Ocelot *vs* Margay | 0.828 | 0.589 | 0.990 | 0.0248 | ns |
|  | Jaguarundi *vs* Margay | 0.337 | 0.121 | 0.587 | 0.1578 | ns |
| VB | Puma *vs* Ocelot | 0.606 | 0.476 | 0.733 | 0.3839 | < 0.01 |
|  | Ocelot *vs* Margay | 0.588 | 0.362 | 0.791 | 0.1114 | ns |
| YAN | Jaguar *vs* Puma | 0.508 | 0.317 | 0.704 | 0.3244 | < 0.01 |
|  | Jaguar *vs* Ocelot | 0.497 | 0.317 | 0.686 | 0.4877 | < 0.001 |
|  | Puma *vs* Ocelot | 0.659 | 0.451 | 0.835 | 0.0563 | ns |
|  | Ocelot *vs* Jaguarundi | 0.314 | 0.164 | 0.475 | 0.7318 | < 0.001 |
|  | Ocelot *vs* Margay | 0.635 | 0.368 | 0.876 | 0.0725 | ns |
|  | Jaguarundi *vs* Margay | 0.325 | 0.125 | 0.525 | 0.2159 | < 0.05 |
| YAS | Jaguar *vs* Puma | 0.720 | 0.581 | 0.859 | 0.23 | < 0.05 |
|  | Jaguar *vs* Ocelot | 0.506 | 0.360 | 0.655 | 0.5508 | < 0.001 |
|  | Puma *vs* Ocelot | 0.668 | 0.559 | 0.772 | 0.5311 | < 0.001 |
|  | Ocelot *vs* Jaguarundi | 0.365 | 0.239 | 0.494 | 0.826 | < 0.001 |
|  | Ocelot *vs* Margay | 0.668 | 0.487 | 0.813 | 0.1229 | ns |
|  | Jaguarundi *vs* Margay | 0.202 | 0.059 | 0.340 | 0.6889 | < 0.001 |
