# Supplementary material for: Prey availability and temporal partitioning modulate felid coexistence in Neotropical forests
Source: PLoS One. 2019 Mar 12;14(3):e0213671. doi: 10.1371/journal.pone.0213671 (PMC6413900; doi:10.1371/journal.pone.0213671)
Supplement: S8 Table — (DOCX) [file pone.0213671.s008.docx]

| S8 Table - Coefficient of overlap (Δ1) with confidence intervals (CI lower/CI upper) and Watson’s two-sample test (two-sample *U*2) performed on pairwise comparisons between study sites (ns – non-significant). | | | | | | |
| --- | --- | --- | --- | --- | --- | --- |
| Species | Sites | Coefficient of Overlap | | | Watson's Two-Sample Test | |
|  |  | Δ1 | CI lower | CI upper | U² | P value |
| Jaguar | CAX - CSN | 0.824 | 0.679 | 0.945 | 0.111 | ns |
|  | CAX - COU | 0.650 | 0.490 | 0.796 | 0.288 | < 0.01 |
|  | CAX - YAN | 0.827 | 0.666 | 0.962 | 0.036 | ns |
|  | CAX - YAS | 0.874 | 0.733 | 0.989 | 0.025 | ns |
|  | CSN - COU | 0.731 | 0.579 | 0.859 | 0.225 | < 0.05 |
|  | CSN - YAN | 0.856 | 0.711 | 0.985 | 0.056 | ns |
|  | CSN - YAS | 0.788 | 0.637 | 0.914 | 0.123 | ns |
|  | COU - YAN | 0.731 | 0.544 | 0.886 | 0.148 | ns |
|  | COU - YAS | 0.642 | 0.489 | 0.790 | 0.319 | < 0.01 |
|  | YAN - YAS | 0.838 | 0.672 | 0.972 | 0.031 | ns |
| Puma | CAX - CSN | 0.841 | 0.722 | 0.948 | 0.066 | ns |
|  | CAX - COU | 0.885 | 0.773 | 0.973 | 0.047 | ns |
|  | CAX - VB | 0.727 | 0.572 | 0.855 | 0.229 | < 0.05 |
|  | CAX - YAN | 0.613 | 0.405 | 0.812 | 0.224 | < 0.05 |
|  | CAX - YAS | 0.748 | 0.604 | 0.893 | 0.232 | < 0.05 |
|  | CSN - COU | 0.795 | 0.665 | 0.909 | 0.132 | ns |
|  | CSN - VB | 0.760 | 0.629 | 0.878 | 0.102 | ns |
|  | CSN - YAN | 0.506 | 0.316 | 0.689 | 0.364 | < 0.01 |
|  | CSN - YAS | 0.728 | 0.586 | 0.847 | 0.195 | < 0.05 |
|  | COU - VB | 0.745 | 0.601 | 0.869 | 0.226 | < 0.05 |
|  | COU - YAN | 0.635 | 0.438 | 0.815 | 0.195 | < 0.05 |
|  | COU - YAS | 0.758 | 0.613 | 0.885 | 0.205 | < 0.05 |
|  | VB - YAN | 0.537 | 0.366 | 0.698 | 0.338 | < 0.01 |
|  | VB - YAS | 0.842 | 0.714 | 0.945 | 0.051 | ns |
|  | YAN - YAS | 0.552 | 0.380 | 0.721 | 0.333 | < 0.01 |
| Ocelot | BCI - CAX | 0.826 | 0.709 | 0.928 | 0.081 | ns |
|  | BCI - CSN | 0.866 | 0.792 | 0.929 | 0.139 | ns |
|  | BCI - COU | 0.876 | 0.825 | 0.924 | 0.126 | ns |
|  | BCI - MAN | 0.866 | 0.792 | 0.929 | 0.138 | ns |
|  | BCI - VB | 0.774 | 0.661 | 0.876 | 0.109 | ns |
|  | BCI - YAN | 0.835 | 0.740 | 0.915 | 0.141 | ns |
|  | BCI - YAS | 0.818 | 0.750 | 0.881 | 0.202 | < 0.05 |
|  | CAX - CSN | 0.819 | 0.684 | 0.925 | 0.123 | ns |
|  | CAX - COU | 0.795 | 0.680 | 0.894 | 0.128 | ns |
|  | CAX - MAN | 0.727 | 0.510 | 0.874 | 0.071 | ns |
|  | CAX - VB | 0.849 | 0.736 | 0.942 | 0.031 | ns |
|  | CAX - YAN | 0.819 | 0.705 | 0.912 | 0.092 | ns |
|  | CAX - YAS | 0.837 | 0.725 | 0.924 | 0.070 | ns |
|  | CSN - COU | 0.796 | 0.728 | 0.861 | 0.369 | < 0.01 |
|  | CSN -MAN | 0.758 | 0.543 | 0.849 | 0.092 | ns |
|  | CSN - VB | 0.772 | 0.668 | 0.873 | 0.192 | < 0.05 |
|  | CSN - YAN | 0.739 | 0.645 | 0.830 | 0.300 | < 0.01 |
|  | CSN - YAS | 0.811 | 0.731 | 0.881 | 0.318 | < 0.01 |
|  | COU - MAN | 0.739 | 0.544 | 0.841 | 0.065 | ns |
|  | COU - VB | 0.783 | 0.671 | 0.879 | 0.228 | < 0.05 |
|  | COU - YAN | 0.836 | 0.745 | 0.903 | 0.058 | ns |
|  | COU - YAS | 0.860 | 0.794 | 0.917 | 0.160 | ns |
|  | MAN – VB | 0.716 | 0.529 | 0.864 | 0.093 | ns |
|  | MAN – YAN | 0.670 | 0.525 | 0.878 | 0.079 | ns |
|  | MAN - YAS | 0.712 | 0.547 | 0.849 | 0.085 | ns |
|  | VB - YAN | 0.752 | 0.640 | 0.857 | 0.176 | ns |
|  | VB - YAS | 0.831 | 0.741 | 0.911 | 0.138 | ns |
|  | YAN - YAS | 0.800 | 0.709 | 0.872 | 0.068 | ns |
| Jaguarundi | BCI - CSN | 0.837 | 0.609 | 1.008 | 0.049 | ns |
|  | BCI - COU | 0.701 | 0.451 | 0.898 | 0.041 | ns |
|  | BCI - YAN | 0.690 | 0.425 | 0.904 | 0.100 | ns |
|  | BCI - YAS | 0.951 | 0.730 | 1.110 | 0.015 | ns |
|  | CSN - COU | 0.727 | 0.533 | 0.880 | 0.067 | ns |
|  | CSN - YAN | 0.683 | 0.496 | 0.851 | 0.184 | ns |
|  | CSN - YAS | 0.827 | 0.657 | 0.966 | 0.066 | ns |
|  | COU - YAN | 0.627 | 0.423 | 0.815 | 0.138 | ns |
|  | COU - YAS | 0.720 | 0.508 | 0.899 | 0.045 | ns |
|  | YAN - YAS | 0.655 | 0.437 | 0.850 | 0.152 | ns |
| Margay | CAX - CSN | 0.718 | 0.570 | 0.862 | 0.158 | ns |
|  | CAX - YAS | 0.499 | 0.311 | 0.694 | 0.165 | ns |
|  | CSN - YAS | 0.743 | 0.553 | 0.899 | 0.038 | ns |
