# Supplementary material for: Prey availability and temporal partitioning modulate felid coexistence in Neotropical forests
Source: PLoS One. 2019 Mar 12;14(3):e0213671. doi: 10.1371/journal.pone.0213671 (PMC6413900; doi:10.1371/journal.pone.0213671)
Supplement: S9 Table — (DOCX) [file pone.0213671.s009.docx]

| S9 Table - Differences in the daily activity level (*i. e.,* proportion of hours per day that an animal is active), standard errors (SE), Wald test (W) of Neotropical cats across the eight study sites (*Significant difference <0.05). | | | | | |
| --- | --- | --- | --- | --- | --- |
| Species | Site | Difference | SE | W | *p* |
| Jaguar | CAX-COU | 0.06 | 0.13 | 0.25 | 0.62 |
|  | CAX-CSN | 0.21 | 0.15 | 2.12 | 0.15 |
|  | CAX-YAN | 0.18 | 0.14 | 1.64 | 0.20 |
|  | CAX-YAS | 0.01 | 0.13 | 0.01 | 0.91 |
|  | COU-CSN | 0.28 | 0.13 | 4.32 | 0.04* |
|  | COU-YAN | 0.25 | 0.13 | 3.69 | 0.05* |
|  | COU-YAS | 0.08 | 0.11 | 0.48 | 0.49 |
|  | CSN-YAN | 0.03 | 0.15 | 0.05 | 0.83 |
|  | CSN-YAS | 0.20 | 0.13 | 2.22 | 0.14 |
|  | YAN-YAS | 0.17 | 0.13 | 1.71 | 0.19 |
| Puma | CAX-COU | 0.09 | 0.14 | 0.45 | 0.50 |
|  | CAX-CSN | 0.01 | 0.13 | 0.01 | 0.91 |
|  | CAX-VB | 0.17 | 0.13 | 1.64 | 0.20 |
|  | CAX-YAN | 0.11 | 0.14 | 0.61 | 0.44 |
|  | CAX-YAS | 0.12 | 0.14 | 0.71 | 0.40 |
|  | COU-CSN | 0.11 | 0.12 | 0.76 | 0.38 |
|  | COU-VB | 0.08 | 0.13 | 0.39 | 0.53 |
|  | COU-YAN | 0.20 | 0.13 | 2.29 | 0.13 |
|  | COU-YAS | 0.03 | 0.13 | 0.04 | 0.85 |
|  | CSN-VB | 0.19 | 0.12 | 2.44 | 0.12 |
|  | CSN-YAN | 0.09 | 0.12 | 0.57 | 0.45 |
|  | CSN-YAS | 0.13 | 0.13 | 1.11 | 0.29 |
|  | VB-YAN | 0.28 | 0.13 | 4.67 | 0.03* |
|  | VB-YAS | 0.05 | 0.13 | 0.17 | 0.68 |
|  | YAN-YAS | 0.22 | 0.13 | 2.78 | 0.10 |
| Ocelot | BCI - CAX | 0.11 | 0.11 | 1.04 | 0.31 |
|  | BCI - CSN | 0.06 | 0.09 | 0.40 | 0.53 |
|  | BCI - COU | 0.11 | 0.07 | 2.84 | 0.09 |
|  | BCI - MAN | 0.25 | 0.11 | 5.66 | 0.02* |
|  | BCI - VB | 0.23 | 0.09 | 6.30 | 0.01* |
|  | BCI - YAN | 0.14 | 0.09 | 2.75 | 0.10 |
|  | BCI - YAS | 0.22 | 0.07 | 10.03 | 0.00* |
|  | CAX - CSN | 0.17 | 0.12 | 1.94 | 0.16 |
|  | CAX - COU | 0.00 | 0.10 | 0.00 | 0.99 |
|  | CAX - MAN | 0.14 | 0.13 | 1.21 | 0.27 |
|  | CAX - VB | 0.13 | 0.12 | 1.07 | 0.30 |
|  | CAX - YAN | 0.03 | 0.12 | 0.09 | 0.77 |
|  | CAX - YAS | 0.11 | 0.10 | 1.17 | 0.28 |
|  | CSN - COU | 0.17 | 0.09 | 3.87 | 0.05* |
|  | CSN -MAN | 0.31 | 0.12 | 6.74 | 0.01* |
|  | CSN - VB | 0.29 | 0.11 | 7.27 | 0.01* |
|  | CSN - YAN | 0.20 | 0.10 | 3.86 | 0.05* |
|  | CSN - YAS | 0.28 | 0.09 | 9.84 | 0.00* |
|  | COU - MAN | 0.14 | 0.10 | 2.00 | 0.16 |
|  | COU - VB | 0.12 | 0.09 | 2.01 | 0.16 |
|  | COU - YAN | 0.03 | 0.08 | 0.17 | 0.68 |
|  | COU - YAS | 0.11 | 0.06 | 3.23 | 0.07 |
|  | MAN – VB | 0.02 | 0.12 | 0.03 | 0.87 |
|  | MAN – YAN | 0.11 | 0.12 | 0.92 | 0.34 |
|  | MAN - YAS | 0.03 | 0.10 | 0.10 | 0.75 |
|  | VB - YAN | 0.09 | 0.10 | 0.78 | 0.38 |
|  | VB - YAS | 0.01 | 0.09 | 0.02 | 0.89 |
|  | YAN - YAS | 0.08 | 0.08 | 0.89 | 0.35 |
| Jaguarundi | BCI - CSN | 0.09 | 0.11 | 0.66 | 0.42 |
|  | BCI - COU | 0.02 | 0.11 | 0.04 | 0.84 |
|  | BCI - YAN | 0.04 | 0.10 | 0.12 | 0.72 |
|  | BCI - YAS | 0.00 | 0.11 | 0.00 | 0.98 |
|  | CSN - COU | 0.11 | 0.10 | 1.16 | 0.28 |
|  | CSN - YAN | 0.12 | 0.09 | 1.75 | 0.19 |
|  | CSN - YAS | 0.09 | 0.10 | 0.84 | 0.36 |
|  | COU - YAN | 0.01 | 0.10 | 0.02 | 0.88 |
|  | COU - YAS | 0.02 | 0.10 | 0.03 | 0.85 |
|  | YAN - YAS | 0.03 | 0.09 | 0.12 | 0.72 |
| Margay | CAX - CSN | 0.08 | 0.10 | 0.71 | 0.40 |
|  | CAX - YAS | 0.14 | 0.11 | 1.85 | 0.17 |
|  | CSN - YAS | 0.06 | 0.09 | 0.48 | 0.49 |
